# The Scandinavian Fantasy: The Sources of Intergenerational Mobility in Denmark and the US†

**DOI:** 10.1111/sjoe.12219

**Published:** 2016-12-16

**Authors:** Rasmus Landersø, James J. Heckman

**Affiliations:** ^1^ The Rockwool Foundation DK‐1307 Copenhagen Denmark; ^2^ University of Chicago Chicago IL 60637 USA

**Keywords:** Comparative analysis of systems, education, inequality, social mobility, *I*24, *I*28, *I*32, *P*51

## Abstract

This paper examines the sources of differences in social mobility between the US and Denmark. Measured by income mobility, Denmark is a more mobile society, but not when measured by educational mobility. There are pronounced non‐linearities in income and educational mobility in both countries. Greater Danish income mobility is largely a consequence of redistributional tax, transfer, and wage compression policies. While Danish social policies for children produce more favorable cognitive test scores for disadvantaged children, they do not translate into more favorable educational outcomes, partly because of disincentives to acquire education arising from the redistributional policies that increase income mobility.

1

The American Dream is now spoken with a Scandinavian accent. (Washington's Blog, 2014)

## I. Introduction

Policy analysts around the world point to Scandinavia as a model for reducing inequality and promoting intergenerational mobility (see, e.g., Baily, 2016). By conventional measures, social mobility by income is much higher there than in the US.

In this paper, we use rich Danish data to explore the sources of these differences in social mobility. By all accounts, Denmark is a prototypical Scandinavian welfare state. Lessons learned from Danish data apply to Scandinavia more generally.

Our investigation reveals some surprises and apparent contradictions. The literature on Danish social mobility by income is surprisingly sparse and uses only a limited number of measures of income. One contribution of this paper is to demonstrate that the choice of the measure of income used matters greatly in determining the relative social mobility of the US and Denmark.

The standard measure of intergenerational mobility is based on the intergenerational elasticity (IGE): a regression coefficient showing the percentage change in a child's income associated with a percentage change in parental income. We show that estimated IGEs depend greatly on the measure of income used and that estimated IGEs vary with the level of income. US social mobility is low (absolutely and compared to Denmark) for children from high‐income families.

Popular discussions of the benefits of the Scandinavian welfare state point to its generous support of childcare and education relative to the US as major determinants of its greater social mobility. In Denmark, college tuition is free, there is ready access to childcare, pregnancy‐leave policy is generous, and there is virtually universal free pre‐kindergarten. Yet, despite these stark policy differences, the influence of family background on educational attainment is surprisingly similar in the two countries. Levels of intergenerational educational mobility are about the same. At higher levels of family income, educational mobility is lower in both countries.

In both countries, cognitive and non‐cognitive skills acquired by age 15 are more important for predicting educational attainment than parental income. The more child‐generous Danish welfare state produces much more favorable distribution of cognitive skills for disadvantaged Danish children compared to their counterparts in the US. The similarity of the influence of family background on educational attainment in the two countries, despite the more favorable distribution of test scores for Danish disadvantaged children, arises in part from the compression of the wage scale and the generous levels of social benefits that discourage Danish children from pursuing further schooling. In addition, the generosity of the Danish welfare state does not prevent sorting of children into neighborhoods and schools on the basis of family background, which appears to benefit the more advantaged.

Scandinavia invests heavily in child development and boosts the test scores of the disadvantaged. It then undoes these beneficial effects by providing weak labor market incentives. Better incentives to acquire skills would boost Danish educational mobility. Stated differently, the greater incentives to acquire education in the US labor market tend to offset its less favorable investments in the cognitive skills of disadvantaged children. In addition, while the Danish welfare state promotes equality of opportunity compared to the US, many barriers remain. There are large skill gaps between the children of the advantaged and the children of the disadvantaged, during early and late childhood. Residential sorting across neighborhoods and schools is strong.

This paper proceeds in the following way. In Section I., we analyze income mobility in Denmark and the US. We examine the sensitivity of estimated income IGEs to alternative measures of income. We examine the sources of differences in income mobility. We also report non‐parametric estimates of income mobility. In Section Non‐Linear Intergenerational Income Elasticities, we examine the relationship between schooling attainment, measures of family financial resources, cognitive and non‐cognitive skills of children at age 15, family background (education and home environment), and measures of schooling quality. We report surprisingly similar effects of family influence on educational attainment in both societies. We show a link between welfare benefits and educational attainment in Denmark. We discuss the role of neighborhood sorting on child educational attainment. In Section Neighborhood Sorting of Children by Family Background, we qualify our analysis. We conclude in Section IV..

## II. Income Mobility

In this section, we explore alternative measures of intergenerational income mobility. Different measures of income convey very different impressions of social mobility. We show how the levels of transfers, the mapping of education to income, the levels and progressivity of taxation, and income inequality differ between the US and Denmark. All four factors affect estimates of income mobility.

We report estimates of non‐linear (NL) IGEs for both countries. We find different patterns depending on which income measure we consider. Differences favoring Denmark appear at the lowest and the highest levels of income.

### Data

#### US Data

We use two US data sources. We use Panel Study of Income Dynamics (PSID) data for our main analysis of intergenerational income mobility. We measure parental income using a nine‐year average from the child's 7th to 15th year.^1^ Child income is measured as income at ages 34–41 down to ages 30–35 for the 1972–1978 birth cohorts. In our main analysis, we only consider individuals with positive incomes. See Section F in the Online Appendix (https://cehd.uchicago.edu/scandinavian-appendix) for more details.

As the sample size for the PSID data is small (relative to the Danish data), we use the March Current Population Survey (CPS; 1968–2014) from the Integrated Public Use Microdata Series (IPUMS)^2^ when we analyze US income distributions. The sample consists of civilian, non‐institutionalized citizens. We use parents in 1987 and individuals aged 36–38 in 2011.

#### Danish Data^3^

For Denmark, we use the full population register data on the entire cohorts born in 1973–1975. We discard individuals who migrate (or whose parents migrate), individuals for whom we have no identification of the father or mother (around 2 percent), and individuals with negative incomes (averaged over the period where we measure income). Parental income is measured as a nine‐year average from when the child is 7–15 years of age, and the child's income is measured at ages 35–37, 36–38, and 37–39 for the 1975, 1974, and 1973 cohorts, respectively. The full sample size is 166,359, and once we restrict to positive incomes the sample is reduced to 149,190 individual parent–child matches.^4^

**Figure A1:**
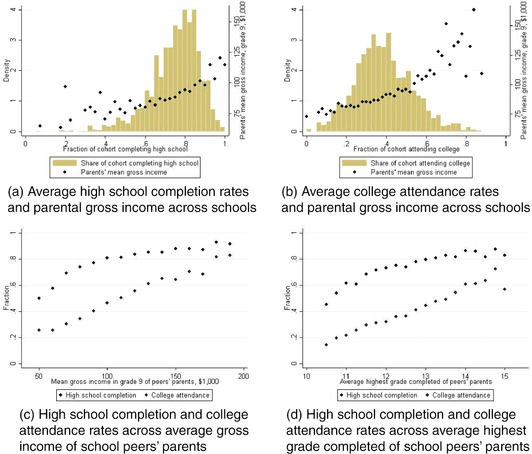
Danish high school completion and college attendance rates, variation across schools, and parental characteristics *Notes*: Panels (a) and (b) show distributions of average high school completion and college attendance rates within a school cohort (born in 1987) in the bars and scatterplots of the bin‐means of parental gross income in ninth grade. Panels (c) and (d) show average high school completion and college attendance rates across the mean levels of the income of peers' (students born in 1987 and belonging to the same school and school cohort) parents. All four plots have been constructed using the Danish register data described in the first subsection of Section Non‐Linear Intergenerational Income Elasticities.

In the Online Appendix, Table A23 provides the definitions of the various income measures we consider, Table A1 summarizes income levels for the US and Denmark by different quantiles and income measures, and Figure A1 depicts the distributions. Table A1 and Figure A1 in the Online Appendix show that incomes in Denmark are more compressed than incomes in the US. There is a large low‐income group in the US that virtually does not exist in Denmark (Forslund and Krueger, 1997; Aaberge *et al*., 2002; Corak, 2013).^5^ In the next section, we show that cross‐sectional differences in income distributions between Denmark and the US are an important source of higher income mobility in Denmark than in the US.

### Linear Intergenerational Income Elasticities

There is a large literature investigating the association between parents' and children's income.^6^ The modal statistic used to study income mobility is the IGE of income $\beta^{IGE}$:

(1) $$\ln\left(Y^{C}\right)=\alpha +\beta^{IGE}\ln\left(Y^{P}\right).$$

The father/son or parent/child IGE is generally found to be much higher in the US than in Denmark. Estimates generally lie between 0.3 and 0.5 in the US and around 0.1 to 0.2 in Denmark (Björklund and Jäntti, 2011; Blanden, 2013; Mazumder, 2005; Solon, 2002). There is a similar range for rank–rank associations. Boserup *et al*. (2013) and Chetty *et al*. (2014) estimate this to be 0.18 in Denmark and 0.34 in the US, respectively. Based on these estimates of the income IGE, Scandinavia is portrayed as a land of opportunity.^7^

Cross‐country differences in estimated IGEs of income can arise for a multitude of reasons that we attempt to capture using different income measures. One measure proxies transmission of total individual income potential with wage earnings, capital income, and profits. Another proxies transmission of total income including public transfers (but not the impact of in‐kind transfers). A third measure introduces the effects of the progressivity of the taxation on income mobility. A fourth measure, wage earnings, proxies intergenerational transmission of earnings‐potential rewarded in labor market – differences arise, in part, from differences in returns to education.

A further source of differences in estimated IGEs arises from differences in levels and trends in cross‐sectional income inequality.^8^ We put this issue aside for now, and investigate it in the following subsection.

Table 1 shows estimated intergenerational income elasticities for similar income measures in Denmark and the US. The odd‐numbered columns report estimates for Denmark. The even‐numbered columns report the corresponding estimates for the US.

**Table 1:** IGE estimates with different income measures: Denmark and the US

	Gross income excl.		Gross income incl.		Wage earnings		Wage earnings and		Net‐of‐tax total
	public transfers		public transfers				public transfers		gross income
	Denmark	US	Denmark	US	Denmark	US	Denmark	US	Denmark
	(1)	(2)	(3)	(4)	(5)	(6)	(7)	(8)	(9)
Estimated IGE	0.352***	0.312***	0.271***	0.446***	0.083***	0.289***	0.063***	0.419***	0.221***
	(0.004)	(0.055)	(0.003)	(0.054)	(0.003)	(0.044)	(0.003)	(0.058)	(0.003)
Observations	149,190	621	149,190	621	149,190	621	149,190	621	149,190

*Notes*: This table shows coefficients ($\beta^{IGE}$) and standard errors from regressions of child log income on parent log income for Denmark and the US. For Denmark, we use full population register data for children born in the period 1973–1975, and for the US we use PSID data for children born in the period 1972–1978. For Denmark, parental income is measured as a nine‐year average from the child's 7th to 15th year, and the child's income is measured at ages 35–37, 36–38, and 37–39 for the 1975, 1974, and 1973 cohorts, respectively. For the US, parental income is measured as a nine‐year average from the child's 7th to 15th year, and the child's income is measured as last‐year income at ages 34–41, 33–40, 32–39, 31–38, 30–37, 30–36, and 30–35 for the 1972, 1973, 1974, 1975, 1976, 1977, and 1978 cohorts, respectively. The columns are based on the following: column 1, for Denmark, all taxable income including wage earnings, profits from own business, capital income, and foreign income excluding all public transfers (both taxable and non‐taxable); column 2, for the US, all taxable income including earnings (payroll income from all sources, farm income, and the labor portion of business income), asset income (such as rent income, dividends, interest, income from trust and royalties, and asset income from business), and private transfers (such as income from alimony, child support, and help from relatives and others); column 3, for Denmark, all taxable income including wage earnings, public transfers, profits from own business, capital income, and foreign income; column 4, for the US, all taxable income including earnings, asset income, private transfers and public transfers (such as social security income, SSI, TANF, ETC, other welfare income, retirement, pension, unemployment, and workers compensation); column 5, for Denmark, taxable wage earnings and fringes, labor portion of business income, and non‐taxable earnings, severance pay, and stock‐options; column 6, for the US, payroll income from all sources (such as wages and salaries, bonus, overtime income, tips, commissions, professional practice, market gardening, additional job income, and other labor income), farm income, and labor portion of business income; column 7, for Denmark, taxable wage earnings and fringes, labor portion of business income, and non‐taxable earnings, severance pay, and stock‐options, plus taxable and non‐taxable public transfers (social assistance, unemployment benefits, labor market leave, sick leave assistance, labor market activation, child benefits, education grants, housing support, early retirement pension, disability pension, and retirement pension); column 8, for the US, payroll income from all sources, farm income, labor portion of business income, and public transfers; column 9, for Denmark, total gross income minus all final income taxes paid in given year (note we do not have information on individual net‐of‐tax income from the PSID). ^+^ *p* < 0.1; *p < 0.05; **p < 0.01; ***p < 0.001.

Column 1 shows that the estimated IGE based on gross income, excluding public transfers, is 0.352 for Denmark. This estimate is much higher than estimates reported in the literature, which use wage earnings, earnings, or income including public transfers. The corresponding estimate for the US is 0.312. The difference between the two estimates is not statistically significant. The third and fourth columns show that the estimated IGE for Denmark drops by around 20 percent to 0.271 when public transfers are included in the measure of income. This decrease illustrates the important role of redistribution in Denmark. For the US, the corresponding estimate jumps to 0.446, bringing us close to the estimate reported in Solon (1992) and Chetty *et al*. (2014) (see Table A1 in the Appendix). Comparing the estimate in column 3 in Table 1 to that of column 9 in the same table, we see that adding taxation reduces the Danish IGE estimate further. Unfortunately, we do not have the data required to estimate the corresponding IGE for the US.^9^

When we focus on wage earnings alone in columns 5 and 6, the estimated IGE for Denmark drops dramatically to 0.083, while the corresponding US estimate is 0.289. Finally, adding public transfers to wage earnings results in an even larger gap between the two countries. For wage earnings plus public transfers, the Danish IGE is 0.063 while the US estimate is 0.419.^10^

Our estimates for Denmark do not contradict the findings of the previous literature. Rather, they enrich our understanding of them. Measured by income potential (columns 1 and 2), we find that intergenerational mobility in Denmark is not significantly different from intergenerational mobility in the US. When we account for public transfers, estimates for the two countries diverge. Income mobility by this measure is substantially higher in Denmark than in the US. When we consider wage earnings alone or wage earnings inclusive of public transfers, we obtain estimates for Denmark reported in the previous literature with estimated IGEs around 0.1.

One should interpret cross‐country differences with great caution. There is no single best measure of the IGE. We do not claim that we have shown that levels of income mobility in the US and Denmark are alike or different. The conclusion from this analysis is that by accounting for transfers, wage compression, returns to education, and progressive income taxation, we can explain a substantial portion of the Denmark–US difference in associations between children's and parents' income.

In addition, several measurement problems discussed in the previous literature (see, e.g., Solon, 2004) might also affect estimated IGEs. Imputing zeros with an arbitrary value affects estimates. Censoring might also produce biased results, for example, by leaving out the long‐term unemployed from the analysis.^11^ Table A5 in the Online Appendix reports the estimates corresponding to Table 1 when imputing zero incomes with $1,000. The table shows that estimated IGEs change for income categories that include many zeros (gross income excluding transfers and wage earnings).^12^ Nevertheless, the overall patterns from Table 1 remain unchanged for Denmark. For the US, however, the PSID data are much more sensitive to the inclusion of zero and non‐reported incomes. In order to obviate the problems with zero income, analyses estimating relationships between children's and parents' ranks in their respective income distributions^13^ have recently been used (see Dahl and DeLeire, 2008; Chetty *et al*., 2014). We do not report results for rank–rank estimates in the main text and we refer readers to the Online Appendix.^14^ ${}^{,}$ 15

### The Role of Inequality in Shaping the IGE

The cross‐country correlation between income mobility and income inequality has received a lot of attention in the past decade (Corak, 2006). Krueger (2012) calls this the “Great Gatsby curve”. In this subsection, we examine the mechanical relationship between estimated IGE and changes in inequality across generations. It follows from the definition of the IGE,

$$\beta^{IGE}=corr\left[\ln\left(Y^{C}\right),\ln\left(Y^{P}\right)\right]\frac{sd[\ln\left(Y^{C}\right)]}{sd[\ln\left(Y^{P}\right)]},$$

that an increase in inequality from one generation to the next amplifies the estimate without affecting mobility measured by correlation coefficients. Hence, differences in inequality between generations and countries might generate differences in perceived income mobility.^16^

Table 2 shows how differences in variances drive estimates. The table shows the regression coefficients from Table 1 together with the correlation and intergenerational ratio of standard deviations below each coefficient. The table shows that, although not statistically significantly different, the intergenerational correlation for gross income excluding transfers in the US is above its Danish counterpart. It is the ratio of standard deviations that drives the Danish IGE to levels above the US. When public transfers are included in gross income, the correlation and ratio increase in the US, while in Denmark the ratio decreases and the correlation is roughly unchanged. These results also emphasize that transfers are more progressive and constitute a larger fraction of income in Denmark compared to the US. Furthermore, the table shows that the large increase in the estimated IGE for the US when public transfers are included, partly arises because transfers reduce inequality in parents' income while inequality in children's income is largely unaffected.^17^ When we focus on wage earnings alone, the correlation in Denmark drops from a level of 0.214 to 0.081, whereas in the US the intergenerational correlation remains unchanged.^18^ Table A5 in the Online Appendix presents a corresponding analysis imputing zero incomes with $1,000. The main difference for Denmark is that intergenerational correlations for gross income excluding transfers and wage earnings increase to 0.246 and 0.118, respectively, while the correlations for the remaining incomes measure remain largely unaffected. Hence, including individuals with zero incomes, there is a substantial reduction in the intergenerational correlation when we add transfers to gross income in Denmark.

**Table 2:** Intergenerational correlations and inequality for different income measures: Denmark and the US

	Gross income excl.		Gross income incl.		Wage earnings		Wage earnings and	
	public transfers		public transfers				public transfers	
	Denmark	US	Denmark	US	Denmark	US	Denmark	US
	(1)	(2)	(3)	(4)	(5)	(6)	(7)	(8)
$\beta^{IGE}$	0.352***	0.312***	0.271***	0.446***	0.083***	0.289***	0.063***	0.419***
$\rho_{Child,Parents}\frac{sd(Child)}{sd(Parents)}$	$0.201\frac{0.860}{0.491}$	$0.268\frac{0.977}{0.840}$	$0.214\frac{0.375}{0.308}$	$0.318\frac{0.906}{0.645}$	$0.081\frac{1.004}{0.989}$	$0.256\frac{0.970}{0.860}$	$0.075\frac{0.561}{0.669}$	$0.280\frac{0.923}{0.615}$

*Notes*: This table shows coefficients ($\beta^{IGE}$) and standard errors from regressions on parental log income on child's log income from Table 1, together with the correlation multiplied with the ratio of the standard errors $\beta^{IGE}=\rho_{Child,Parents}\left(sd(Child)/sd(Parents)\right)$ for Denmark and the US. ^+^ *p* < 0.1; *p < 0.05; **p < 0.01; ***p < 0.001.

From this analysis, we see that IGE estimates are sensitive not only to the income measures used, but also to inequality levels and changes. It is not meaningful to compare IGE estimates, when arbitrary large or small levels of inequality drive the estimates. In order to investigate this issue in greater depth, we conduct a further analysis showing the sensitivity of IGE estimates to adjustments for inequality.

**Figure A2:**
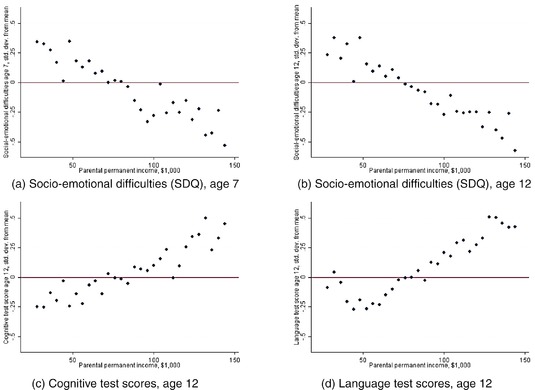
Test score gaps in Denmark at age 7 and age 12, by parental permanent gross income *Notes*: The figure shows deviations of SDQ scores (in panels (a) and (b)), CHIPS scores (cognitive test in panel (c)), and a language test score (in panel (d)) relative to the sample mean by $4,000 bins of parental permanent gross income including transfers. Scores have been standardized to mean 0 and standard deviation 1. A higher score of SDQ implies greater socio‐emotional difficulties. A higher score in the CHIPS and language test implies better cognitive and language skills. Figures have been constructed using Danish register data in combination with the Danish Longitudinal Study of Children (DALSC); see http://gl.sfi.dk/Default.aspx?AreaID=21.

We present regressions where we transform the different income distributions for Denmark to the corresponding income distributions for the US, holding income ranks fixed. Then, we place the US distribution in the Danish distribution. In the upper panel of Table 3, we present IGE estimates where quantiles of the Danish distributions – for parents (reported in the rows) and for children (reported in the columns) – are mapped into the equivalent income measures for the US for children born in the period 1973–1975 in 2011 with parents in the 1987 March CPS data. Figure A2(a) in the Online Appendix illustrates the transformation for wage earnings distributions. The child or parent with the *n*th total gross income rank in Denmark is assigned the total gross income level associated with the *n*th rank of total gross income for the US child or parent distribution. A similar transformation is used for total income excluding public transfers, total net‐of‐tax income, wage earnings, and wage earnings plus public transfers. Using this method, we illustrate what the Danish IGE would be for the different income measures, if Denmark had the same levels of inequality within generations as those found in the US.

**Table 3:** IGE estimates with different income measures, imposing US income distribution on Danish distribution, and vice versa

	Gross income excl.		Gross income incl.		Net‐of‐tax total		Wage earnings		Wage earnings and	
	public transfers		public transfers		gross income				public transfers	
	Baseline	Transformed	Baseline	Transformed	Baseline	Transformed	Baseline	Transformed	Baseline	Transformed
	child	child	child	child	child	child	child	child	child	child
	inc. dist.	inc. dist.^*a*^	inc. dist.	inc. dist.	inc. dist.	inc. dist.	inc. dist.	inc. dist.	inc. dist.	inc. dist.
	(1)	(2)	(3)	(4)	(5)	(6)	(7)	(8)	(9)	(10)
**Panel A: US distribution imposed on Danish distribution**										
Baseline parent	0.352***	0.435***	0.271***	0.600***	0.221***	0.392***	0.083***	0.071***	0.063***	0.104***
	(0.004)	(0.005)	(0.003)	(0.008)	(0.003)	(0.006)	(0.003)	(0.002)	(0.002)	(0.003)
Transformed parent	0.187***	0.230***	0.093***	0.213***	0.093***	0.168***	0.150***	0.138***	0.068***	0.124***
	(0.002)	(0.002)	(0.001)	(0.003)	(0.001)	(0.002)	(0.003)	(0.002)	(0.002)	(0.002)
**Panel B: Danish distribution imposed on US distribution**										
Baseline parent	0.308***	0.207***	0.433***	0.186***			0.302***	0.247***	0.394***	0.177***
	(0.051)	(0.042)	(0.060)	(0.028)			(0.051)	(0.050)	(0.062)	(0.032)
Transformed parent	0.666***	0.501***	0.734***	0.337***			0.338***	0.304***	0.378***	0.186***
	(0.095)	(0.079)	(0.105)	(0.048)			(0.055)	(0.054)	(0.067)	(0.034)

*Notes*: This table shows coefficients ($\beta^{IGE}$) and standard errors from regressions of child log income on parents' log income using the samples, variables, and income definitions detailed in the notes to Table 1. The columns labeled “Baseline child” show IGE of income where child's income distribution is unchanged. The columns labeled “Transformed child” show IGE of income where children's income distribution has been changed to fit the distributions of the corresponding income measures for the US in panel A and the corresponding income measures for Denmark in panel B. Rows labeled “Baseline parent” show IGE of income where parents' household income distribution is unchanged. Rows labeled “Transformed parent” show IGE of income where parents' household income distribution has been changed to fit the reported income distributions for corresponding income measures for the US in panel A and for Denmark in panel B. Number of observations: panel A, 149,190; panel B, 621. ^+^ *p* < 0.1; *p < 0.05; **p < 0.01; ***p < 0.001.

This is a radial transformation quantiles of Danish child income distribution mapped into quantiles of comparable measures of US distribution.

In the lower panel of Table 3, we do the opposite, which is illustrated in Figure A2(b) in the Online Appendix. In this case, quantiles of the US distributions are mapped into the equivalent income measures in 2010–2012 for Denmark for children born in the period 1973–1975 and their parents, using Danish register data. Hence, we illustrate what the US IGE would be for the different income measures, if the US had the same levels of inequality within generations as those found in Denmark.

The columns and rows labeled “Baseline” for both parents and children show the actual IGE coefficients from Table 1. The first line in the upper panel shows that if Danes born between 1973 and 1975 had the same income distribution as the corresponding US age cohorts, the Danish IGE estimates for gross income excluding transfers, including transfers, and net‐of‐tax would increase by 50–100 percent, whereas it would be unchanged for wage earnings. In the next thought experiment, we examine the consequences of giving Danish parents the same income distribution as US parents. Estimated IGEs would decrease. Transforming children's and parents' income distribution reduces IGEs by 30–50 percent when we consider the gross income measures, and increases IGEs by roughly 50–100 percent when we consider wage earnings and wage earnings plus transfers.

When we perform the equivalent exercise for the US, we naturally reach the opposite conclusion. Changing the income distribution of children while holding parents' income distributions fixed results in large reductions in the IGE, whereas changing income distributions for parents while holding children's income distributions fixed results in large increases in the IGE. Finally, by transforming both generations' income distributions, the IGE by gross income excluding transfers increases, the IGE for income measures including transfers decreases, and the IGE for wage earnings is unchanged.

Table 3 shows that IGEs in Denmark and the US are quite different when wage earnings and wage earnings plus transfers are used as measures of income. Estimated IGEs based on these two income measures are robust to the changes in inequality that we observe for both countries. For the remaining measures of income, the similarities between the IGEs in Denmark and the US are substantial and depend strongly on trends and levels in inequality.

The analyses presented in this section emphasize that levels of estimated income (im)mobility depend on the subjective evaluation of the reader. Not only do estimates vary by income measures, they are also affected by whether changing inequality is linked to mobility. For example, with a fixed correlation between children's and parents' income, doubling income inequality from one generation to the next clearly increases differences in income levels and the consumption possibilities between children from high‐income and low‐income families. Should the chosen measure of income mobility capture such change? Without specifying a social welfare function and a normative definition of fairness, this question does not have a clear answer.

### Non‐Linear Intergenerational Income Elasticities

It is likely that any benefits from the Scandinavian welfare states accrue to the least advantaged. This is a feature that linear models of the IGE might fail to adequately capture. Thus, it is particularly interesting to analyze non‐linearities in the IGEs. Wage compression and the high level of redistribution via taxes and transfers only add weight to the relevance of considering possible non‐linearities.

Yet, few previous studies consider non‐linearities. Bratsberg *et al*. (2007) report that the relationship between the logarithm of child and parent income is convex in Denmark (and in Scandinavia more generally) and concave in the US for measures of wage earnings.^19^ They attribute this finding to higher mobility for individuals from low‐income families in Denmark than in the US. We replicate these findings in Figure A3 in the Online Appendix. However, as previously emphasized, results differ according to which income measure is used. In Denmark, for example, wage earnings of children and parents display a convex relationship, while for total gross income excluding public transfers the relationship is linear, or perhaps even concave.

We account for non‐linearities using local linear regressions. We estimate the NL‐IGE, $\beta^{IGE}\left[\ln\left(Y_{0}^{P}\right)\right]$, where $Y_{0}^{P}$ is the income of the parent at *Y*_0_ and $Y^{C}$ is income of the child:^20^

(2) $$\begin{array}{ccc} & & \min_{\alpha \left[\ln\left(Y_{0}^{P}\right)\right],\beta \left[\ln\left(Y_{0}^{P}\right)\right]}\overset{N}{\sum_{i=1}}K_{h_{\lambda }}\left(Y_{0}^{P},Y_{i}^{P}\right)\\ & & \;\times {\left\{\ln\left(Y_{i}^{C}\right)-\alpha \left[\ln\left(Y_{0}^{P}\right)\right]-\beta^{IGE}\left[\ln\left(Y_{0}^{P}\right)\right]\ln\left(Y_{i}^{P}\right)\right\}}^{2}. \end{array}$$

It is feasible to estimate the NL‐IGEs using absolute income, thereby obviating the problem that ln(0) does not exist.^21^ However, the estimation of NL‐IGE using absolute income involves a trade‐off in terms of precision for high income levels due to the right tail of the income distribution. Doing so reduces the precision of estimates substantially. In order to be able to compare estimates from $10,000 to $125,000 and not just from $30,000 to $60,000 of parental incomes, we therefore report estimates using log income here. The corresponding point estimates using absolute income are very similar to the results shown in the main text and are reported in Figures A5 and A6 in the Online Appendix.

**Figure 1:**
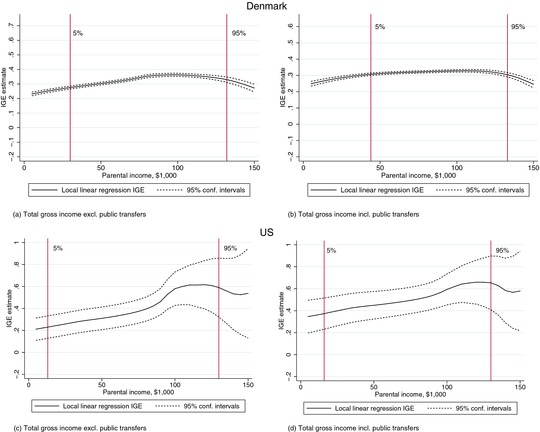
Local intergenerational income elasticity in Denmark and the US *Notes*: The figures show estimated intergenerational income elasticities of wage income plus public transfers for Denmark (panels a and b) and the US (panels c and d). Panels (a) and (b) have been constructed using full population register data from Denmark, and panels (c) and (d) have been constructed using PSID data. The figures show local linear regression slopes of log of children's income on log of parental income. Local linear regressions are weighted using kernels of absolute income. Standard errors for panels (a) and (b) have been constructed from 50 bootstraps, and standard errors for panels (c) and (d) have been constructed from 1,000 bootstraps. The vertical lines indicate the 5th and 95th percentiles in the respective income distributions.

**Figure 2:**
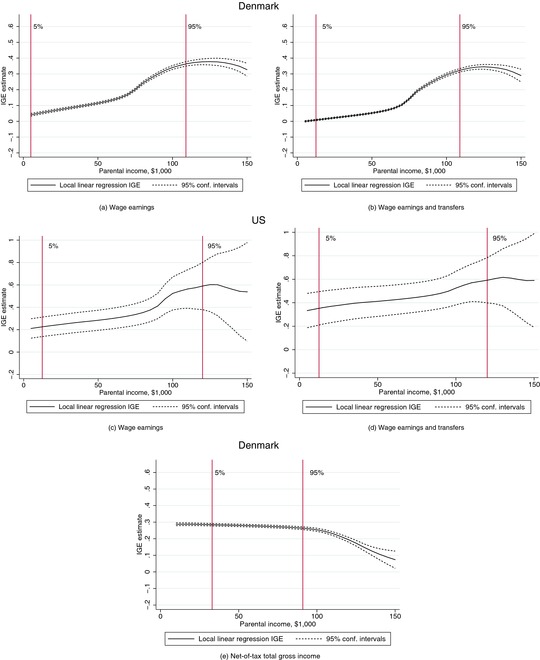
Local intergenerational income elasticity in Denmark and the US (continued) *Notes*: The figures show estimated intergenerational income elasticities of wage income plus public transfers for Denmark (panels (a), (b), and (e)) and the US (panels (c) and (d)). Panels (a), (b), and (e) have been constructed using full population register data from Denmark, and panels (c) and (d) have been constructed using PSID data. The figures show local linear regression slopes of log of children's income on log of parental income. Local linear regressions are weighted using kernels of absolute income. Standard errors for panels (a), (b), and (e) have been constructed from 50 bootstraps, and standard errors for panels (c) and (d) have been constructed from 1,000 bootstraps. The vertical lines indicate the 5th and 95th percentiles in the respective income distributions.

Figures 1 and 2 show plots of NL‐IGE estimates of log income, weighted with absolute income, without imputation for zero income for Denmark and the US. The vertical lines in the figures mark the 5th and 95th percentiles in the income distributions in the Danish data and the 5th and 95th percentiles for the US data. It should be noted that these estimates only allow us to infer local conclusions about mobility. A zero IGE estimate at a low level of income does not imply that going from rags to riches is likely. It only shows that a marginal movement up (or down) in income levels relative to parental income is just as likely as the status quo. The figures present estimates for the income ranges where the data allow us to make meaningful estimates (because there is very limited support for high incomes in the PSID).

Figure 1 shows NL‐IGE estimates for gross income excluding public transfers and gross income including public transfers for Denmark and the US (Figures 1(a) and 1(b)). The elasticity goes from levels around 0.25 to almost 0.4 when parental income increases from $0 to $100,000. Thereafter, the estimates slowly decline and reach a level of around 0.1–0.2 at the 99th percentile of parental income. The corresponding results for the US (in Figures 1(c) and 1(d)) show that elasticities at low income levels closely correspond to those in Denmark, although they are imprecisely estimated. In the US, elasticities increase monotonically with parental income. At the 95th percentiles of parental gross income excluding and including public transfers, US intergenerational income elasticities are well above 0.5.

Figure 2 graphs NL‐IGE estimates for wage earnings and wage earnings plus public transfers for Denmark (panels a and b) and the US (panels c and d), and net‐of‐tax total gross (disposable) income for Denmark (panel e). Figures 2(a) and 2(b) show functional forms similar to those from Figures 1(a) and 1(b). Elasticities at low levels of income are approximately 0.1, or even lower, and increase monotonically until parental income reaches $110,000. After this point, the elasticities decrease. For the US, the figures also show a monotonic upward slope but with levels well above the Danish estimates at all points. Finally, elasticities for net‐of‐tax income in Denmark are initially flat at roughly 0.25 but go towards zero as parental income increases beyond the 99th percentile.

**Figure 3:**
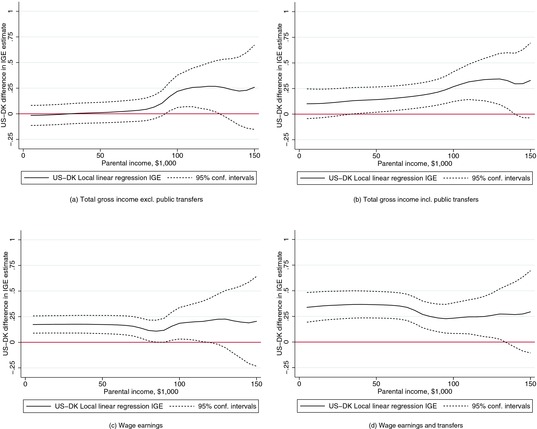
US–Denmark difference in local intergenerational income elasticity *Notes*: This figure shows the US–Denmark difference in local intergenerational income elasticities from Figures 1 and 2. The figure shows the US IGE estimate minus the Danish estimate, such that a positive value indicates a larger IGE estimate for the US than for Denmark and a negative value indicates the opposite.

In order to obtain a more precise view of the cross‐country differences in NL‐IGEs, Figure 3 plots the differences between the US and the Danish elasticities across levels of parental income. From Figure 3(a), we see that income mobility in gross income excluding public transfers is roughly similar for family incomes up to $100,000. From this point onward, a gap emerges that – albeit imprecisely estimated – continues to increase. Income mobility by gross income excluding transfers is much lower in the US than in Denmark for top quartile family incomes, but not for families with low income. When transfers are added to income, as shown in Figure 3(b), the elasticities in the US are persistently above the Danish elasticities with a widening gap at high incomes. When we only consider wage earnings in Figure 3(c), the Danish IGE is around 0.2 lower than the US IGE across all parental income levels. This result also dovetails nicely with our argument about the importance of wage compression in Denmark, as opposed to the increasing return to education in the US, being key mechanisms behind the observed income mobility differences. Finally, Figure 3(d) shows that for wage earnings plus transfers, intergenerational income elasticities in Denmark are consistently below US levels. Here, the Danish IGE is around 0.35 lower than the US IGE at low income levels and 0.25 lower at high income levels. Hence, the largest difference between income mobility in Denmark and the US is now for the lowest family incomes.

## III. Educational Mobility by Family Background

In the previous section, we studied intergenerational income mobility across two countries and show that wage compression and tax/transfer policies are major determinants of cross‐country differences in mobility. Although the reward for education might be lower in Denmark, its generous support of education, support of childcare, and early education initiatives promote skill formation as measured by test scores among the disadvantaged.

Many point to the more generous educational and childcare policies in place in Denmark as a source of its greater social mobility (e.g., Sanders, 2013). We examine this claim and show that average educational mobility is remarkably similar across the two countries. We start by demonstrating the near‐universal participation in such programs in Denmark coupled with a lack of educational and income gaps compared to the US.^22^

**Figure 4:**
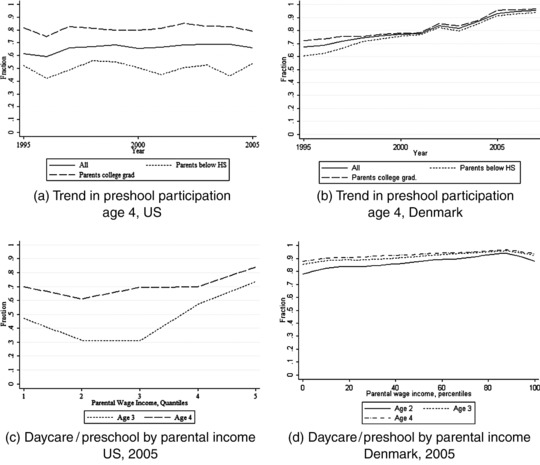
Daycare and preschool use *Notes*: Panels (a) and (b) show the fractions enrolled in preschool at age 4 from 1995–2007 for all children and by parents' education. Panels (c) and (d) show the fractions in daycare/preschool by parental wage income rank within child cohort in 2005. The US figures are constructed from October CPS data, and the Danish figures are constructed from administrative register data.

Figures 4(a) and 4(b) show the fraction of children enrolled in preschool programs at the age of 4 in the US and Denmark from 1995 to 2005.^23^^,^ 24 The figures show the overall rates, together with the rates for children for whom both parents have fewer than 12 years of schooling (less than high school) and for children for whom both parents have at least 15 years of schooling (college or more). The figures show that average preschool enrollment rates at age 5 were, on average, similar in the two countries in 1995. Since then, rates of participation have stagnated in the US and increased to a level close to full uptake in Denmark. Importantly, the figures also show large gaps in enrollment rates by parental education in the US, whereas there are no differences in Denmark.

Figures 4(c) and 4(d) show rates of daycare/preschool use at ages 2, 3, and 4 by parental wage income rank in 2005 in the two countries. Enrollment rates are lower in the US, trends in participation are flatter, and family income gradients for participation in the programs are steeper.

A few studies present causal evidence linking access to universal public childcare to improvements in skills in a Scandinavian context. Havnes and Mogstad (2011b) study a large expansion of childcare in Norway on long‐run outcomes. They find that daycare enrollment improves educational attainment and earnings, especially for children from low‐resource families.^25^ Datta Gupta and Simonsen (2010, 2012) investigate the effects of home care, non‐parental/related family care (i.e., in a child‐minder's home), and public daycare in Denmark on socio‐emotional skills. Datta Gupta and Simonsen (2010) find that public daycare relative to family care increases socio‐emotional skills at age 7, while Datta Gupta and Simonsen (2012) suggest that the effects might fade at later ages.

There is an active body of literature in which the effectiveness of early childhood interventions in the US is investigated.^26^ The evidence from many US programs might not be relevant to the current discussion, as the programs are often very intensive and target specific groups of children. Cascio (2009) reports that large‐scale, publicly funded childcare programs in the US are less effective than their Scandinavian counterparts. She suggests that low‐intensity programs crowd out other programs (e.g., Head Start) and divert funding from other public skill formation initiatives.

**Figure 5:**
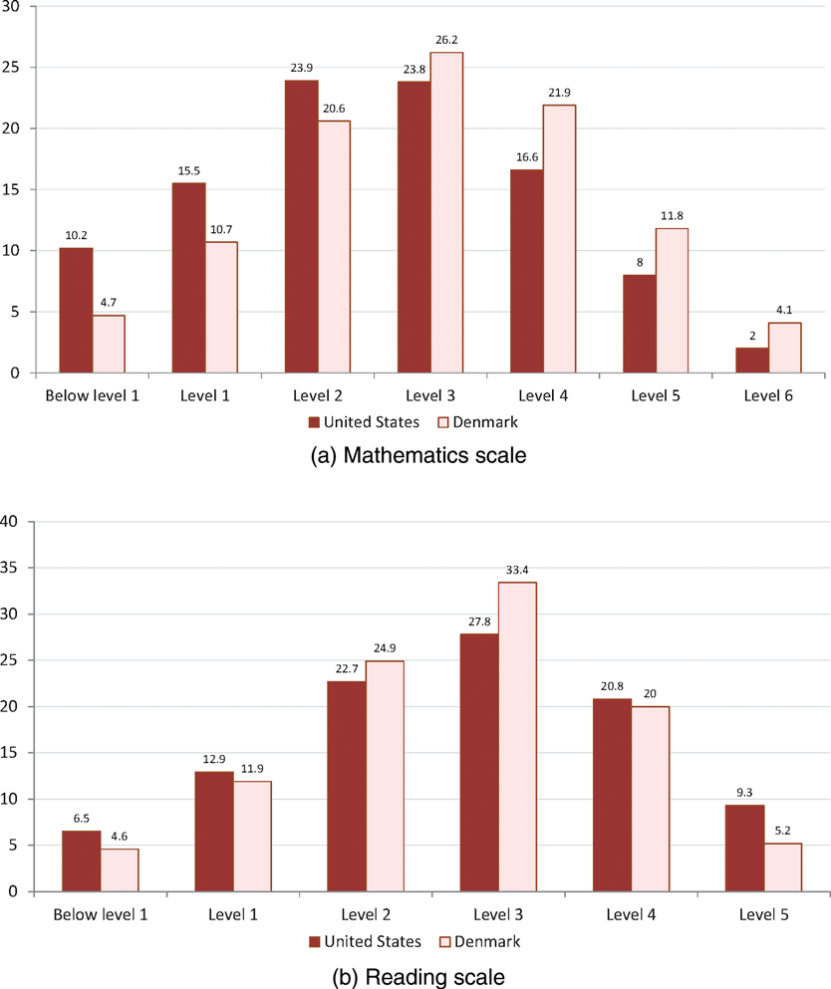
Percentage of students at each proficiency level, PISA 2003 *Source*: OECD (2004).

While the cited studies only investigate policy changes within a given country, they support the claim that increased early childhood investments, through universal public childcare, improve the skills of the least advantaged children and thus intergenerational skill mobility. The evidence for their effectiveness is supported by Figure 5, which shows distributions of Program for International Student Assessment (PISA) mathematics and reading scores in Denmark and the US in 2003.^27^ The figure shows stark differences in the lower tails of PISA test scores. The lowest quartile in the US performs much worse than the lowest quartile in Denmark.

**Figure 6:**
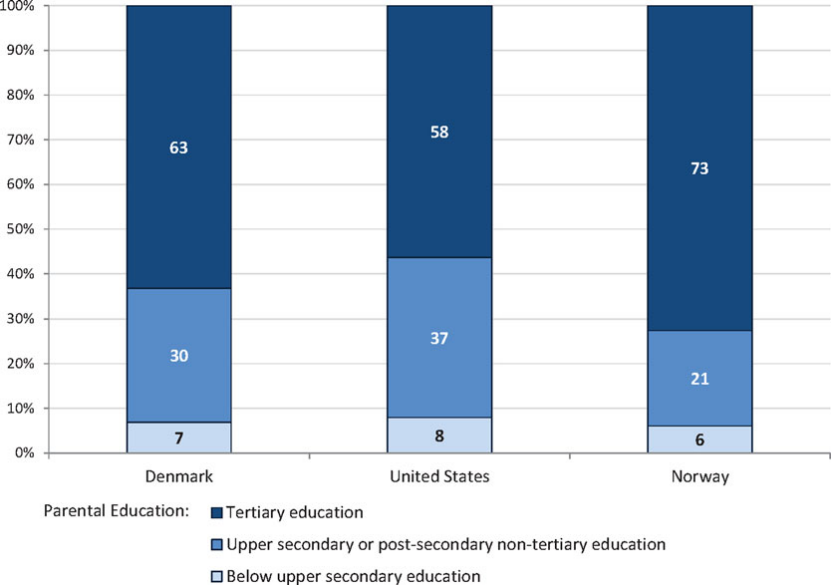
Proportion of those aged 20–34 in tertiary education, by parents' educational attainment, for Denmark, Norway, and the US *Notes*: This figure shows the proportion of those aged 20–34 in tertiary education, by parents' educational attainment (below upper secondary, upper secondary or post‐secondary (non‐tertiary), and tertiary education) in 2012, for Denmark, Norway, and the US. *Source*: OECD (2014, Chart A4.1).

Yet, despite the greater provision of early childhood education to low‐resource families in Denmark, the lack of any pecuniary costs of education in Denmark, the compressed skill distributions, and the association between educational attainment levels from one generation to the next are remarkably similar in Denmark and the US. Figure 6 shows the fraction of those aged 20–34 in (or with) a tertiary education, by parental educational attainment in Denmark, the US, and Norway. The figure shows that only 6–8 percent of individuals aged 20–34 who are enrolled in or have completed a tertiary education come from homes where both parents have not graduated from an upper secondary education. Generally, there are few differences in these percentages across the three countries. Figure A13 and Section B.2 (Figure A30) in the Online Appendix corroborate this evidence. Figure A13 shows that educational mobility in Denmark is not higher than in the US if we instead consider regression‐based coefficients relating children's and parents' educational attainment as reported in Hertz *et al*. (2008). Educational transitions across generations are very similar in the two countries for more recent cohorts, as we show in Figure A30 in the Online Appendix.

In the rest of this section, we elucidate these findings and investigate the reasons why seemingly similar levels of educational mobility arise. First, we briefly describe the data used in our analyses. Then, we examine educational attainment by parental resources and which factors help to explain the relationship between children's education and parents' resources. We also consider explanations that link the findings from our different analyses.

### Data

#### US Data: CNLSY

We restrict the sample to cohorts born in 1991 or earlier.^28^ In addition to information on own characteristics, we include information on mother's characteristics from the original National Longitudinal Survey of Youth (NLSY) data. We restrict the sample to individuals for whom we observe at least one test score for both cognitive and non‐cognitive skills (see below), along with parental income. This leaves us with a sample of 3,268 individuals. We lose 15 percent because of missing observations, and 28 percent of the remaining sample are born in 1987 or later. See Table A19 in the Online Appendix for sources of loss of sample information.

#### Danish Data: 1987 Cohort

We use the entire cohort of children born in Denmark in 1987. Using a unique individual identifier, we link information on demographic characteristics to schooling outcomes and exam grades in ninth grade.^29^ The data also include a unique parental identifier, which allows us to link the information on the children to parental income and wealth, demographic characteristics, and mother's educational attainment. We restrict the sample to children whose parents have non‐negative household wage income in 2002. This results in a sample of 39,539 children.^30^

#### Comparability of Samples

There are two fundamental differences between the Danish and US samples. First, while the latter come from survey data, the former come from a full population register based on information reported from relevant institutions and authorities. Second, cohorts vary in their characteristics in the two countries. Danish data are centered around a child's birth year. The data from the Children of the National Longitudinal Survey of Youth (CNLSY) are centered around parents' birth year, as these data are based on children born from five cohorts of parents. In the CNLSY, we record information on multiple cohorts of children (before and after 1987) and only five cohorts of mothers. For the Danish data, we consider only one cohort of children born in 1987 and numerous cohorts of parents. We do not censor the data to align parents' and children's cohorts between the two countries, as this would induce heavy selection in terms of mother's age at childbirth. As female fertility patterns are different between the US and Denmark, such selection imposes arbitrary differences between the two countries and could consequently invalidate the analysis.^31^

#### Measuring Income and Wealth

In the CNLSY data, we measure parental income using the sum of the mother's and the spouse's self‐reported wage earnings. In the Danish data, we measure parental income as the sum of the mother's and father's wage earnings.^32^ For both countries, we measure income as average income between the child's 3rd and 15th years. The two income concepts are similar in content.

For the US, we measure assets by reported net assets in the CNLSY.^33^ For Denmark, assets are measured by net assets (excluding pension savings) from income and wealth data reported to tax authorities.^34^ In both countries, we measure assets at age 15 of the child. While the data again differ in terms of source, net assets are highly dependent on housing wealth.^35^ Thus, intra‐country differences in wealth can capture both differences in market luck in the housing sector, family endowments, and lifetime income.^36^

#### Measuring Education

In the US data, high school completion is defined using questions on whether or not the child has a high school diploma/General Educational Development certificate (GED).^37^ We define college attendance as a report of either full‐ or part‐time enrollment in college. In the Danish data, we define high school completion as having completed an education that requires at least 12 years of schooling, which includes both academic and vocational high school graduates, and college as having been enrolled in an education that requires at least 15 years of schooling.^38^

Cross‐country institutional differences are a potential confounder. While we have chosen our definitions of high school completion and college attendance to maintain comparability, we do not (and cannot) control for all cross‐country institutional differences. Two potentially problematic issues are social promotion^39^ and the minimum school leaving age,^40^ which might distort the levels of human capital associated with equal levels of schooling in Denmark and the US.

#### Measuring Skills

For the US, we use the Peabody Individual Achievement Test (PIAT) scores to measure cognitive skills. The CNLSY features three sets of PIAT scores: reading recognition, reading comprehension, and mathematics. For non‐cognitive skills, we use the antisocial, headstrong, hyperactivity subscales from the Behavior Problem Index (BPI). The measures of cognitive skills and non‐cognitive skill are in accordance with those of, for example, Cunha and Heckman (2008) and Heckman *et al*. (2006).

For Denmark, we measure skills using grades from the ninth grade. Cognitive skills measured are residualized by non‐cognitive measures. Exam grades (even cognitive ones) are highly dependent on non‐cognitive skills (the final year of compulsory schooling, i.e., before they begin high school; Borghans *et al*., 2016). We measure cognitive skills using final mathematics exam grades (written), mathematics mid‐term grades (written), final physics exam grades, and non‐cognitive skills using orderliness/organization/neatness grades from the Danish written exam, Danish written mid‐term, and mathematics written exam.^41^ As test scores and grades are highly associated with non‐cognitive skills (Borghans *et al*., 2011a,b, 2016), we use residuals from the cognitive measures regressed on the non‐cognitive measures in the measurement system to estimate cognitive skills.

### Education and Family Background

Figures A14(a)–(d) in the Online Appendix show children's educational attainment measured by completion of high school or equivalent (Figures A14(a) and (b)) and enrollment into college or equivalent (Figures A14(c) and (d)) by the log of parental wage income and wealth in the US and Denmark.^42^

Figures A14(a) and (b) show that, in both countries, rates of high school completion increase in parental income and wealth. In the US data, the relationship has its greatest curvature at low levels of income and wealth, while a gradient is evident across the entire range of parental wealth and income in the Danish data. In both countries, 90 percent of children at the top of the income and wealth distribution complete high school, whereas for low levels of income and wealth, approximately 65–70 percent complete high school in the two countries. Broadening the income and wealth ranges to all levels of support beyond the ranges where we have an overlap between the two countries, Figure A15 in the Online Appendix shows that individuals whose parents are at the lower end of the distributions are more likely to complete high school in the US than in Denmark. For parents with low levels of income and wealth, 60 and 45 percent of children in the US and Denmark, respectively, complete high school.

Figures A14(c) and (d) show that college attendance rates also increase with parental wealth and income. The gradient, with respect to wealth, is larger in the US than in Denmark at the bottom of the wealth distribution. Parental income is only strongly associated with increasing rates of college attendance for families with above‐median wealth in the US. In contrast, in Denmark, the income gradient is largest for families with below‐median wealth.^43^

Finally, Figures A19(a) and (b) in the Online Appendix show level differences of the surfaces displayed in the previous figures for the areas of income and wealth where we have common support in the Danish and US data. The figures show that levels of high school completion are higher in the US than in Denmark for children from low‐income/low‐wealth families, while this group's college attendance rates are substantially higher in Denmark than in the US.

The figures just described illustrate that levels of social mobility in Denmark do not always exceed social mobility in the US. One result in Figure A14 might suggest that mobility is higher in Denmark while another suggests the opposite. We next investigate which mediating factors explain mobility (or lack thereof) in Denmark and the US, and whether the role of these factors differ.

### Controlling for Skills Formed in Early Adolescence, Family Characteristics, and Sorting of Children into Schools by Parental Income

In this subsection, we adjust the figures discussed in the previous subsection by controlling for cognitive and non‐cognitive skills, measures of family background, and measures of school characteristics. Doing so significantly reduces the income and wealth differentials, with early adolescent measures of cognitive and non‐cognitive skills playing a major role.

Table 4 presents linear regression estimates of parental log income and wealth on children's high school completion and college attendance for the US and Denmark. The estimates can thus be interpreted as elasticities.^44^ In the upper panel of the table, we present estimates with high school completion as the outcome; in the lower part of the table, we present results with college attendance as the outcome. Below each set of estimates for Denmark and the US, we show differences in coefficients between the two countries and the associated *p*‐values for tests of differences. The first column of the table shows the unadjusted linear estimates corresponding to Figure A14 in the Online Appendix, and in the subsequent three columns we gradually increase the conditioning set.^45^

**Table 4:** Regression coefficients for high school completion and college attendance on parental resources by different conditioning sets

	(1)	(2)	(3)	(4)
**US, high school completion**				
Parental permanent wage income age 3–15	0.033***	0.023**	0.017*	0.006
	(0.009)	(0.008)	(0.008)	(0.007)
Parental wealth (net assets) age 15	0.020***	0.018***	0.015***	0.012***
	(0.003)	(0.003)	(0.003)	(0.003)
**Denmark, high school completion**				
Parental permanent wage income age 3–15	0.066***	0.050***	0.045***	0.002
	(0.003)	(0.002)	(0.002)	(0.002)
Parental wealth (net assets) age 15	0.037***	0.025***	0.023***	0.002
	(0.001)	(0.001)	(0.001)	(0.001)
**Difference in slope: US–Denmark**				
Δ Parental permanent wage income age 3–15	−0.033	−0.027	−0.028	0.004
*p*‐value	0.001	0.001	<0.001	0.583
Δ Parental wealth (net assets) age 15	−0.017	−0.007	−0.008	0.010
*p*‐value	<0.001	0.027	0.001	0.002
**US, college attendance**				
Parental permanent wage income age 3–15	0.063***	0.041***	0.035***	0.019**
	(0.010)	(0.010)	(0.010)	(0.009)
Parental wealth (net assets) age 15	0.022***	0.019***	0.010***	0.008**
	(0.003)	(0.004)	(0.004)	(0.003)
**Denmark, college attendance**				
Parental permanent wage income age 3–15	0.061***	0.043***	0.037***	0.011**
	(0.003)	(0.003)	(0.003)	(0.003)
Parental wealth (net assets) age 15	0.034***	0.018***	0.015***	0.001
	(0.001)	(0.001)	(0.001)	(0.001)
**Difference in slope: US–Denmark**				
Δ Parental permanent wage income age 3–15	0.002	−0.002	−0.002	0.008
*p*‐value	0.848	0.774	0.848	0.400
Δ Parental wealth (net assets) age 15	−0.012	0.001	−0.005	0.007
*p*‐value	<0.001	0.998	0.134	0.058
Residualing by:				
$\theta^{C},\theta^{NC}$		X	X	X
Family background			X	X
School characteristics				X

*Notes*: The table shows regression coefficients of parental permanent wage income and wealth on children's high school completion and college attendance for increasing conditioning set with skills, family background, and school characteristics. The table is constructed using data from the CNLSY for the US/administrative register data on the full cohort born in 1987 for Denmark. The table shows *p*‐values from tests of equal slope‐coefficients against a two‐sided alternative. Observations: US, 3,268; Denmark, 39,539. ^+^ *p* < 0.1; *p < 0.05; **p < 0.01; ***p < 0.001.

From column 1 of the table, we see that parental income and wealth are strongly associated with children's high school completion and college attendance in the US and in Denmark. As shown in Figure A14, parental income and wealth gradients for children's high school completion are significantly higher in Denmark, while only the gradient for wealth differs for college attendance.

The second column presents associations controlling for child level of cognitive and non‐cognitive skills measured at age 15–16. In comparison to the estimates from the first column, the income and wealth gradients for high school completion and college attendance are substantially reduced. Thus, the relationship between parental resources and child education is to a large degree mediated by levels of cognitive and non‐cognitive skills in the adolescent years. While the upper panel shows that the coefficients for income and wealth still differ between Denmark and the US for high school completion, the estimates in the second column of the lower panel show that there are no significant differences between Denmark and the US for either income or wealth gradients in college attendance.

Even though cognitive and non‐cognitive skills are highly predictive of educational attainment in both Denmark and the US, cross‐country differences in these skills do not explain the entire relationship between parental resources and educational attainment. When we extend the analysis in the third column by adding measures of parental background (education/family status) to the measures of child skills, the relationship between parental financial resources and child education weakens further.^46^ Again, we find that associations between parental income and wealth, on the one hand, and children's probability of high school completion, on the other, are stronger in Denmark compared to the US. We find no cross‐country differences in the estimated relationships for college attendance.

Cognitive and non‐cognitive skills and parental/family background play similar roles in mediating the relationship between parental financial resources and children's educational outcomes in both countries. However, other differences remain in comparing educational income and wealth gradients in the two countries. For example, Denmark and the US differ in the variability of school quality. Differences between the quality of public and private schooling likely depend on overall resources devoted to public schools – a major difference between the two countries. Denmark spends a far greater fraction of its GDP on public education than the US.^47^ Yet, school resources and peer characteristics still vary by parental resources in Denmark, suggesting similar relationships between measures of schooling quality and family characteristics across the two countries. We present a preliminary exploration of these relationships for Denmark later in this section, where we establish that a school quality gradient also exists in Denmark. However, because of the lack of data, we are unable to test for differences in the distributions of school quality.

In the final column of Table 4, we show estimates of the association between children's education and parental income and wealth conditioning on the child's level of skills at ages 15–16, family background measures, and school characteristics measured in the primary school years. The gradients in parental income and wealth are substantially reduced because quality measures for primary school predict later educational attainment.^48^ For Denmark, there is no remaining statistically significant relationship between parental resources and children's education, while for the US, a small relationship remains. Moreover, we only find one statistically significant cross‐country difference at a 5 percent level between the gradients of children's rates of high school completion and college attendance as functions of parental financial resources, and generally none of the estimates differ qualitatively between Denmark and the US.

### Non‐Linear Elasticities between Children's Education and Parents' Income

**Figure 7:**
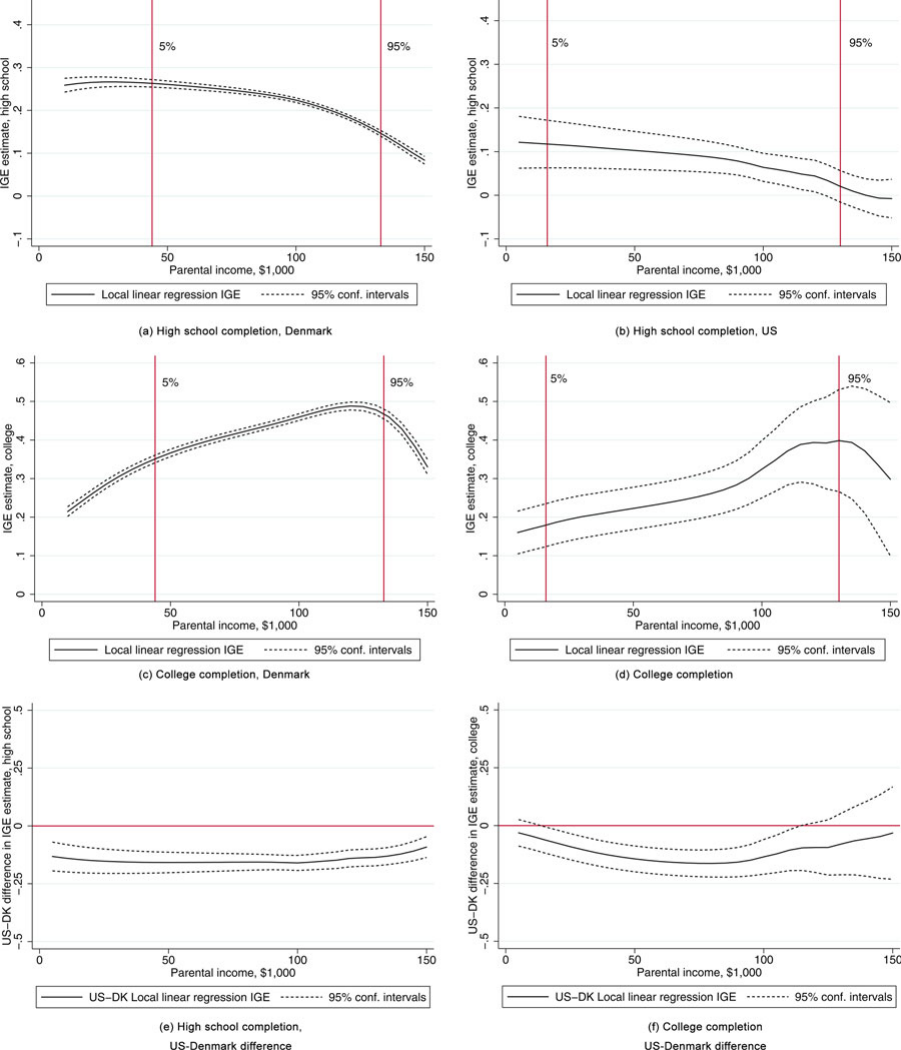
Local intergenerational elasticities between children's education and parental log gross income including transfers, absolute income weights: Denmark and the US *Notes*: Panels (a)–(d) show local linear regression slopes of children's education (high school completion, college graduation) on log of parental gross income including transfers for Denmark and the US. Panels (e) and (f) show US–Denmark differences in local intergenerational elasticities between children's education and parental log gross income including transfers. High school completion is defined as highest completed grade ⩾12, and college graduation as highest completed grade ⩾15. Local linear regressions are weighted using kernels of absolute income. Standard errors are constructed from 50 and 1,000 bootstraps, respectively. The vertical lines mark the 5th and 95th percentiles in the data.

The results reported in Table 4 are average estimates for the two populations in question. As we have argued for income mobility, it is likely that any benefits from the Scandinavian welfare states accrue to the least advantaged. To allow for non‐linearities in the association between children's education and parent's gross income including transfers, we repeat the analysis from Section I. and estimate local linear regressions of children's high school completion/college graduation on parents' log income. Using the same data and estimation strategy as used in Section I., Figures 7(a)–(d) examine the non‐linearities in the elasticities between children's high school/college completion and parental gross income including transfers. Figures 7(e) and 7(f) show the cross‐country differences between the estimated elasticities.^49^

The figures show strong non‐linearities within countries and across educational levels. Elasticities for high school completion vary between 0 and 0.3 in Denmark, and between 0 and 0.12 in the US. For college graduation, the non‐linear relationship with parental income is even more apparent. In both countries, elasticities vary between approximately 0.10–0.15 for low‐income families and 0.40–0.45 for families with an average annual income of around $125,000. Yet, as shown in Figures 7(e) and (f), there is no substantial difference in educational mobility between Denmark and the US. When differences arise, they often do not favor Denmark. Moreover, the shape of the cross‐country differences in educational mobility across parents' total gross income does not show any strong non‐linear pattern favoring the least advantaged in Denmark relative to the least advantaged in the US.

These results also shed light on the likely relationship between credit constraints in the adolescent years and educational attainment, which is investigated by a large body of literature.^50^ This literature is often inconclusive as it does not control for the other parental characteristics associated with income. A related strand of literature investigates the effects of tuition and restrictions to funding of education.^51^ Even though we do not explicitly address this issue, our results are consistent with the evidence that it is not income *during the adolescent years* that matters, but investments as crystallized in cognitive and non‐cognitive skills and longer‐term family background factors that drive these associations.^52^ Our analysis shows that most of the association between high school completion and parental resources, and around half of the association between college enrollment and parental resources, is accounted for by differences in children's cognitive and non‐cognitive skills and family background in early adolescence.^53^ Although the US and Denmark constitute two opposite poles in terms of tuition costs, the income and wealth gradients in high school completion and college enrollment do not differ substantially between the two countries.

In conclusion, despite the higher cognitive scores for the disadvantaged and the lower pecuniary costs of education in Denmark, our analysis in this section together with several other supplementary data sources (see Sections A.2 and B.4 in the Online Appendix; OECD, 2014; Hertz *et al*., 2008) all point in the same direction. There are few noteworthy differences in educational mobility between the US and Denmark, and certainly nothing that can explain the differences in income and wage earnings mobility reported in Section I.. This analysis raises the following important question. Are there factors embedded in the Scandinavian welfare state that reduce incentives to pursue education and thus educational mobility? We discuss and investigate this in the next subsection.

### Welfare Levels and Educational Incentives

It is well established that the economic returns to education are substantially lower in Denmark and the other Scandinavian countries than in the US (e.g., Harmon *et al*., 2003; Fredriksson and Topel, 2010). Two mechanisms leading to this difference are wage compression and the high levels of welfare benefits observed in Scandinavia. As noted in Edin and Topel (1997) and Fredriksson and Topel (2010), incentives to pursue education diminish as returns to education decrease and welfare benefits increase. In this subsection, we establish an empirical relationship between educational attainment and potential public benefits in Denmark. We refer the reader to Section B in the Online Appendix, and to Edin and Topel (1997), Fredriksson and Topel (2010), Freeman *et al*. (2010), Rosen (1997), and Tranæs (2006) for discussions and descriptive evidence of the differences between the income and employment prospects of unskilled or low‐skilled individuals in Denmark and the US, and the relationship between public sector employment, public benefits, and the wage floor in Denmark. In Section B in the Online Appendix, we further show that incomes are compressed in the tails of the educational and income distributions in Denmark. Hence, the lower returns to education in Denmark compared to the US do not stem from cross‐country differences in educational tracks, which could cloud the relationship between years of schooling and income.

**Figure 8:**
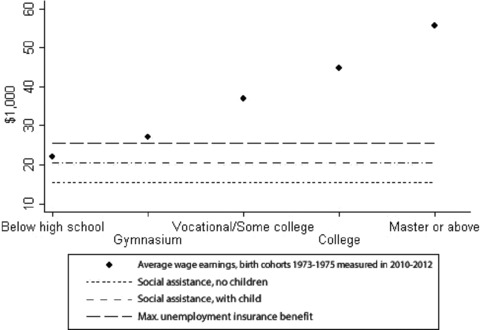
Average wage earnings and potential benefits levels in 2010–2012 for cohorts born in 1973–1975 in Denmark *Notes*: This figure shows average wage earnings per year from 2010 to 2012 for the cohorts born in 1973–1975 in Denmark. The figure also shows maximum unemployment insurance benefits, social assistance level for individuals with children (extra benefit for second child applies), and social assistance level for individuals without children. The horizontal lines are the raw benefits and do not include means‐tested daycare slots and other types of benefits. Average wage earnings are estimated from the full sample and are not conditional on employment. Education: “Below high school” is years of schooling <12; “Gymnasium” is defined as 12 years of schooling and a gymnasium or HF degree (see discussion of HF in footnote 36); “Vocational/Some college” is defined as 12< years of schooling <15, or 12⩽ years of schooling <15 and a vocational training degree; “College” is defined as 15⩽ years of schooling <17; “Master or above” is defined as years of schooling ⩾17, which corresponds to at least a masters degree from a university. Wage earnings include taxable wage earnings and fringes, labor portion of business income, and non‐taxable earnings, severance pay, and stock‐options.

Figure 8 presents evidence on the issue at hand. The figure shows mean pre‐tax wage earnings measured in 2010–2012 for the cohorts born in the period 1973–1975 in Denmark, by level of highest completed education,^54^ together with horizontal lines indicating the 2011 maximum unemployment insurance benefits and the social assistance levels in Denmark.^55^ From the figure, it is evident that for individuals with the lowest levels of education, average wage earnings barely exceed maximum social assistance levels. Even as one climbs the educational ladder, it is not until college completion that wage earnings are twice the size of earnings from social assistance. The progressivity of the Danish tax system only makes this pattern more pronounced.

**Figure 9:**
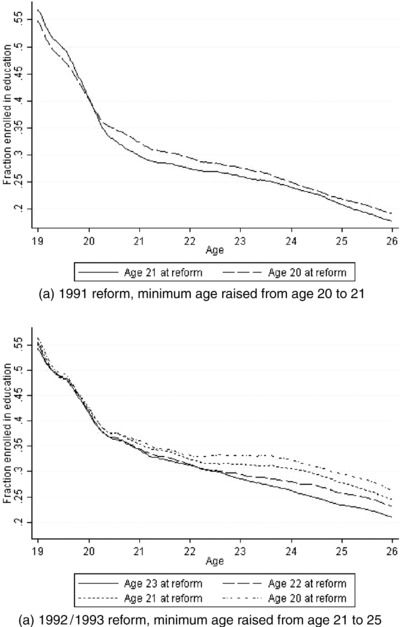
Fraction enrolled in education by age around the timing of two reforms in 1991 and 1992/1993 that raised the minimum age for eligibility for full social assistance levels, in Denmark *Notes*: This figure shows the fraction enrolled in an education by age (measured weekly) from age 19 until age 26 for the cohorts born in 1969 and 1970, and between 1971 and 1974, respectively. The figures are constructed using full population register data with exact enrollment and exit dates from all educational levels (except first to seventh grades) in Denmark, merged with demographic registers with exact information of birth dates. Both panels show enrollment rates around reforms where the minimum age for full social assistance eligibility was raised. In 1991, it was raised from age 20 to age 21. In 1992/1993, it was raised further from age 21 to age 25. Below this age of eligibility, the level of social assistance was substantially reduced and increased activation obligations applied. Panel (a) shows enrollment rates for individuals who were 20 and 21 when the age for full social assistance eligibility was raised from 20 to 21. Panel (b) shows enrollment rates for individuals who were between 20 and 23 when the age for full social assistance eligibility was raised from 21 to 25.

While the relationship between returns to education, public benefits, and educational attainment has been discussed in the literature we have cited, there is little causal evidence. Figure 9 provides the first evidence of such a causal relationship by illustrating the response to two reforms of social assistance levels for youths passed in Denmark in 1991 and 1992/1993, respectively, which increased the incentive to be enrolled in education relative to dropping out.^56^ The first reform raised the minimum age of eligibility for full social assistance (SA) from age 20 to age 21 and the second reform raised the minimum age for receipt from 21 to 25. Below the minimum age, individuals were only entitled to “youth assistance” (*ungdomsydelse*), which was substantially lower than the full SA level, and they had an increased obligation to participate in employment‐focussed activation programs.^57^

Figure 9 shows rates of enrollment in any level of education measured on a weekly basis from age 19 to age 26 for the cohorts born in the period 1969–1974. In Figure 9(a), we plot enrollment rates for individuals who were 20 and 21 years old at the timing of the 1991 reform that raised the minimum age from 20 to 21. The figure shows that enrollment rates were similar at younger ages, but at the exact timing of the reform the two groups diverged and enrollment rates became approximately 2–3 percentage points higher for the affected group who were 20 years old at the timing of the reform, relative to the unaffected group who were 21 years old.

Figure 9(b) shows a similar response around the timing of the 1992/1993 reform that raised the minimum age from age 21 to 25. The figure shows enrollment rates for individuals who were 20–23 years old at the timing of the reform. We see that the groups had similar trajectories prior to the change but diverged once the minimum age was raised. Those affected at age 20 broke away from the three remaining cohorts at age 20. Those affected at age 21 broke away from the two remaining cohorts at age 21. Those affected at age 22 diverged at this exact age from those who were affected at age 23.

The results presented here establish a negative relationship between educational enrollment and the level of public benefits, albeit with two caveats. First, it is beyond the scope of this paper to estimate the underlying behavioral parameters – we strongly encourage future research to explore this relationship. Second, we have neither precise estimates of the potential gains from the greater equality in childhood investments and fewer pecuniary costs of education in Denmark than in the US, nor the disincentives for educational attainment that wage compression and public benefits constitute. Hence, we cannot determine whether the similarities in educational mobility in the two countries occur because the effects offset each other, although we find this to be a plausible explanation given the evidence.

### Neighborhood Sorting of Children by Family Background

**Figure 10:**
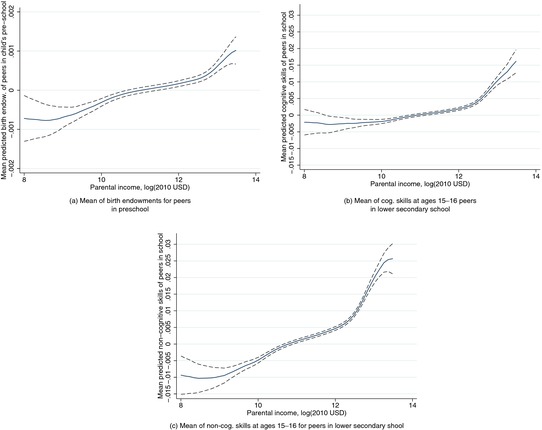
Peers' skill levels across parental income rank, Denmark *Notes*: This figure shows school “leave‐one‐out” means of predicted cognitive and non‐cognitive skills, from the estimated measurement system, using the cohort born in 1987 in Denmark. Mean of peers' birth endowments in preschool are calculated using the 1995 birth cohort. The dashed lines show 95 percent confidence intervals. The skills are anchored to P(high school completion) and the *y*‐axis can be interpreted as such. Hence, a difference of 0.02 from the log(income) of 10 to log(income) of 11 for non‐cognitive skills implies that the mean level difference in non‐cognitive skills for peers of children whose parents' log(income) equal 10 and 11, respectively, are associated with a 2 percentage point difference to the likelihood of completing high school. We use birth weight, gestational length, and length at birth to estimate birth endowments. We use exam grades on mathematics and physics to estimate cognitive skills and grades on organization/neatness to estimate non‐cognitive skills.

**Figure 11:**
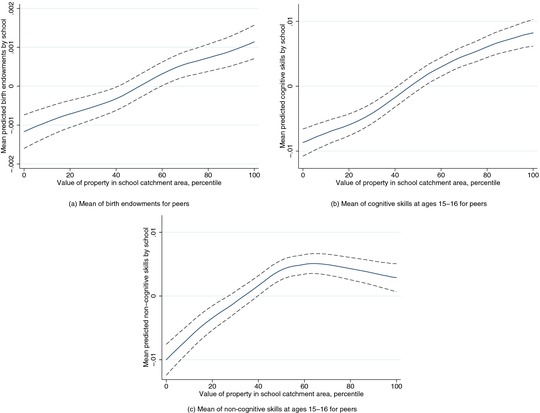
Peers' skill levels across property value in catchment area of lower secondary school, Denmark *Notes*: This figure shows school “leave‐one‐out” means of predicted birth endowments and cognitive and non‐cognitive skills, from the estimated measurement system, using the cohort born in 1987 in Denmark. The dashed lines show 95 percent confidence intervals. The skills are anchored to P(high school completion) and the *y*‐axis can be interpreted as such. Property value is measured as mean valuation of owned property (from Statistics Denmark and Danish national tax authorities) in a given catchment area. Hence, a difference of 0.02 from the 1st to the 100th percentile for cognitive skills implies that the mean level of cognitive skills for peers of children whose parents own property in the most expensive school catchment area are associated with a 2 percentage point higher likelihood of completing high school. We use birth weight, gestational length, and length at birth to estimate birth endowments. We use exam grades on mathematics and physics to estimate cognitive skills and grades on organization/neatness to estimate non‐cognitive skills.

Neighborhood sorting by family socio‐economic status is prevalent in each country. Public schooling in Denmark is universal and attempts to offer all children equal amounts of high‐quality schooling. This policy might be disequalizing because children with early advantages accumulate skills at a higher rate while in school.^58^ High levels of equal investments in schooling for all children amplify initial gaps between advantaged and disadvantaged children. This is a consequence of static complementarity between investments and child skill levels at each age, reinforced by increasing complementarity between investments and skill levels as children age.^59^ Figures 10 and 11 show that, in Denmark, different measures of parental resources correlate with the school and peer quality of public schools,^60^ and thus investments in children through the public schools tend to increase with parental income.^61^

In a similar vein, Figures A1(a) and (b) show variation in average high school completion and college attendance rates across schools. In some schools, only 50 percent of students complete high school and 10 percent of students attend college, respectively, while in other schools, all students complete high school and 80 percent attend college. The figures also show large differences in average parental gross income across schools. The differences correlate strongly with the later educational attainment of students. Figures A1(c) and (d) plot the average high school completion and college attendance rates against the school mean peer parental gross income and highest grade completed. The figures show that the average educational attainment of a ninth grade student is strongly positively correlated with peer family income and education.

Catchment areas for public institutions that limit peers to sort with certain income groups and equal public investments tend to favor children from high‐income families.^62^ The strong relationship in Denmark between educational attainment and family background could arise solely as a result of neighborhood sorting on the basis of family income and wealth.

We lack comparable information for the US. If, in fact, sorting is equally strong in the two countries, this factor helps to explain the near equality of educational IGEs in both countries. Residential sorting might help to undo the benefits of the Scandinavian welfare state. We leave this topic for future research.

Certainly, full equality of opportunity is not present in Denmark. Figure A2 shows socio‐emotional ratings measured at age 7 and 12, and cognitive and language test scores measured at age 12 by parental permanent gross income for Danish children. For all three measures, the average gaps between the most disadvantaged children and the most advantaged children are around 0.5 of a standard deviation. Thus, as evidenced by this figure and our analysis earlier in this section, substantial skill gaps throughout childhood and adolescence remain in Denmark. While the Scandinavian welfare state invests heavily in children throughout childhood and redistributes income (consumption) during adulthood, it has not eradicated the strong influence of parents and early childhood environments. As a consequence of the complementarity between skills and investments, later life universal schooling investments during childhood or adolescence might be ineffective in reducing gaps between advantaged and disadvantaged children.

## IV. Limitations, Future Directions, and Open Questions

Before concluding, we discuss some limitations of this study. First, like much of the empirical literature on social mobility, we report empirical relationships (and not necessarily causal relationships) across generations. Our discussion emphasizes the need for a clearer theoretical framework to disentangle the effects of different income sources (wage earnings, profits, capital, public transfers, and taxation) and mechanisms through which they are related across generations (the dynamics of parental and public investments/human, monetary, and physical capital transmission).

Second, our analysis measures parental income as permanent income during a child's primary and secondary schooling ages. Permanent family income over the life cycle of children has been shown to account for most of the variation in the relationship between family income and children's schooling (Carneiro and Heckman, 2002). Yet, it might be that the pivotal differences between the US and Denmark materialize at early ages.^63^ Low‐income parents might be constrained in making early lifetime investments in the US but not in Denmark.^64^

Third, any attempt to capture a country's level of intergenerational mobility and the relationship between parental and child outcomes by a few point estimates is bound to be unsatisfactory. While the estimation of NL‐IGEs is a step in the right direction, other empirical strategies might be used. One strategy estimates local rank regressions, where the IGE is found by minimizing the product of ranked residuals, thus putting less weight on extreme observations and more weight on mid‐rank observations. Our empirical analysis of rank regressions is consistent with our analysis of NL‐IGEs. There is curvature in the estimated relationships at the top and at the bottom of the income distribution.^65^ Another method is copulas, which might be particularly useful in the present case of describing the dependence between parental and child income because tail dependence in income distributions is notoriously difficult to determine.^66^

There are a number of aspects of inequality that we have not analyzed. We have not addressed issues pertaining to in‐kind transfers, health, and access to health care, but only to inequality in terms of skill formation, educational attainment, and income. A more comprehensive analysis would be desirable.

## V. Conclusion

Academics and policymakers around the world point with admiration to Scandinavia as a model for reducing inequality and promoting social mobility without sacrificing economic efficiency or growth. This paper takes a first step towards investigating in what dimensions and for what reasons Scandinavia is more effective in promoting social mobility.

Despite Denmark's far more generous welfare state, its extensive system of preschools, and its free college tuition, the family influence/child education relationship is very similar to that of the US. In both countries, much of the average association between parental resources and the educational attainment of children can be explained by factors set in place by age 15, including child skills. However, distributions of cognitive test scores of disadvantaged Danish children are much better than those of their counterparts in the US.

The failure to promote greater educational mobility in spite of providing generous social services is most likely rooted in the welfare state. Our findings point to wage compression and the higher levels of welfare benefits as being counterproductive in providing incentives to pursue education. The low returns to education observed in Denmark help to explain the disconnect between the egalitarian childhood policies in Denmark and the roughly equal levels of educational mobility in Denmark and the US. The sorting of families into neighborhoods and schools by levels of parental advantage is likely to be another contributing factor. While the Danish welfare state might mitigate some childhood inequalities, substantial skill gaps still remain.

While patterns of educational attainment are similar across the two countries, the relationships linking skills and income differ greatly. The IGE estimates of income mobility – used as evidence for Scandinavia's high social mobility – are very sensitive to the choice of income measure analyzed. Using total income potential excluding public transfers as a measure of income, there are fewer differences between estimated IGEs for Denmark and the US than previously portrayed. Considering wage earnings or wage earnings plus public transfers, average income mobility is higher in Denmark than in the US. We find evidence of strong non‐linearities in measures of intergenerational income mobility. Differences in Danish–US income mobility favor Denmark (i.e., produce lower local IGEs) at higher levels of parental income and at very low levels of parental income. The education–family background gradients are also non‐linear in both countries but do not favor Denmark at either tail of the parental income distribution.

This paper sends a cautionary note to the many enthusiasts endorsing the Scandinavian welfare state. We make no statements about the *optimality* and *fairness* of the US and Danish systems from a philosophical or social choice point of view. The Danish welfare state clearly boosts the cognitive test scores of disadvantaged children compared to their US counterparts. However, test scores are not the whole story, or even the main story of child success, despite the emphasis on them in popular discussions. Moreover, substantial gaps in test scores remain across social groups within Denmark.

Differences in income mobility between Denmark and the US also arise from wage compression in the Danish labor market, the progressivity of the Danish tax‐transfer system, and the increasing college premium in the US and the rise in inequality there. These factors drive the higher population average income mobility in Denmark and equalize post‐tax consumption possibilities. They also discourage educational attainment in Denmark. Along with neighborhood sorting, they explain the similarity in the influence of family background on educational attainment in the two countries.

The US excels in incentivizing educational attainment. The Danish welfare state promotes cognitive skills for disadvantaged children. Policies that combine the best features of each system would appear to have the greatest benefit for promoting intergenerational mobility in terms of both income and educational attainment.

## Footnotes

1 Throughout the analysis, we use total parental income and do not adjust for differences in household size by the number of siblings.

2 All calculations are weighted by CPS sampling weights and are deflated using the personal consumption expenditure (PCE) deflator.

3 Throughout the paper, we use a purchasing power parity (PPP) adjusted exchange rate of US $100 to 776 Danish Kroner (DKK), as reported by the OECD.

4 Table A2 presents results where we sample cohorts in the Danish register data with the same distribution as observed in the PSID data, and where we sample the number of observations in each cohort as observed in the PSID data. The results do not differ significantly or qualitatively from our main results, which we present in Table 1.

5 See Section B in the Online Appendix. Freeman *et al*. (2010) discuss a broad range of likely causes and consequences of wage compression for the Swedish welfare state. See also Aaberge *et al*. (2000), Pedersen and Smith (2000), and Tranæs (2006), who provide similar evidence from Denmark.

6 See, for example, Blanden (2013), Corak (2006), and Solon (2002) for reviews of the literature.

7 See Table A1 in the Appendix to this paper for a summary of previous IGE estimates for Denmark (comprehensive) and the US (selected).

8 The previous literature investigating social mobility has long addressed some of the issues. One early example is Solon (1992).

9 Table A3 in the Online Appendix shows IGE estimates while controlling for a child's highest completed grade. The table shows that controlling for own education reduces the IGE estimates by approximately one‐third relative to the unadjusted estimates presented in Table 1. Yet, the qualitative differences between income measures and countries remain unchanged. IGE estimates are similar for Denmark and the US for gross income excluding transfers, but diverge for other income measures. In addition, it is evident from the table that the coefficients for a child's highest completed grade on income are larger in the US than in Denmark. Furthermore, the coefficients for a child's highest completed grade for Denmark decrease substantially when we consider income measures including transfers or post‐tax income, whereas they are unaffected by the inclusion of transfers for the US.

10 In Table A4 in the Online Appendix, we show the corresponding IGE estimates while controlling for parents' education. The estimated elasticities decrease by 25–30 percent, but we find no sign of any patterns or cross‐country differences that are not present for the unadjusted IGE estimates in Table 1.

11 Additional measurement problems include life‐cycle bias and measurement error from year‐to‐year variation in income. We attempt to avoid these potential biases by considering parental and child income measured as averages over several years (permanent income) and by measuring children's income when they are in their late 30s.

12 For Denmark, estimated IGEs increase the smaller number used to make the imputation. When we use $1,000, the estimated IGE for gross income excluding transfers is 0.49, and when we use $1 it increases to around 0.6.

13
$\beta^{RR}\left(rank-rank\right)=\mathrm{corr}\left[R\left(Y^{P}\right),R\left(Y^{C}\right)\right]$, where R(*) denotes children's and parents' ranks in their respective distributions. While $\beta^{RR}$ is scale‐invariant in income, $\beta^{IGE}$ is not. The link between the two measures depends on the underlying distributions (see Trivedi and Zimmer, 2007).

14 Table A6 in the Online Appendix replicates this analysis for rank–rank regressions. The findings are qualitatively similar. For total gross income excluding transfers, the Danish estimates are close to the US levels reported by Chetty *et al*. (2014, Table 1, line 7, column 1). When we consider wage earnings alone and/or include public transfers, large cross‐country differences arise (compare Danish levels to Chetty *et al*., 2014, Table 1, line 8, column 1).

15 Rank–rank analyses do not solve the issues that the researcher faces when using log income. We refer the reader to Section C in the Online Appendix for a discussion. Section C in the Online Appendix presents further results on two of the additional issues often discussed in the previous literature on income mobility. For a recent review, see Black and Devereux (2011). The first issue is life‐cycle bias (i.e., that associations between children's and parents' income will be understated if children's income is measured early in their working career, where yearly earnings do not reflect lifetime earnings). We show that the rank–rank slopes for Denmark do not stabilize until the child's income is measured during their late 30s; see Figure A42 in the Online Appendix and also see Nybom and Stuhler, 2015 for similar evidence from Sweden. We also illustrate that measuring parental income earlier in the child's life reduces the rank–rank slopes. (In Denmark, measuring children's income during their early 20s actually results in negative coefficients.) The second issue is attenuation bias (measurement error bias) that stems from the noise arising from including too few years of income data (Solon, 1992). In the Danish data, this can be much larger than the levels reported in Chetty *et al*. (2014). When we measure parental income when the child is below 10 years of age and add income data from subsequent years to the analysis, the differences in rank–rank slopes based on one and five years of data, respectively, range from 12 to 32 percent, depending on which income measure is used (see Figure A43). However, when we use income measured during the child's late teens and add data from preceding years, the corresponding one‐ to five‐year differences are around 0–3 percent, in accord with the analysis of Chetty *et al*. (2014).

16 In a similar vein, one might question how differential trends in educational inequality affect comparisons across countries with high rates of high school and college degrees in earlier generations, as in the US, and countries where high school and college degrees become modal only over the past 30–50 years, as in Denmark and Norway. This remains an open question.

17 This is also shown in Figure A24 in the Online Appendix, where we use CPS data for the US and register data for Denmark to plot average wage earnings and wage earnings plus transfers in the two countries across different educational levels for the cohorts born in the period 1947–1978.

18 In Tables A8–A12 in the Online Appendix, we report the intergenerational correlations and standard deviations of all major income components for Denmark. The tables show that the increased ratio of standard deviations from wage earnings to gross income stems from capital income and profits from own businesses. The ratio of standard deviations changes drastically for gross income because the covariance between wage earnings and profits is negative for parents and zero for children, thus reducing the overall variance of parents' income relative to children's income.

19 Björklund *et al*. (2012), Chevalier *et al*. (2013), and Davies *et al*. (2005) also study non‐linearities.

20 Estimates closely resemble the slopes found in the plots of log of child and parental income in Figures A3 and A4 in the Online Appendix.

21
$K_{h_{\lambda }}\left(Y_{0}^{P},Y_{i}^{P}\right)$ is an Epanechikov kernel. One should note that it is important to distinguish between a kernel with absolute income $K_{h_{\lambda }}\left(Y_{0}^{P},Y_{i}^{P}\right)$ and a kernel with log income $K_{h_{\lambda }}\left[\ln\left(Y_{0}^{P}\right)\right],\left[\ln\left(Y_{i}^{P}\right)\right]$. The former assigns symmetric weight around $Y_{0}^{P}$ while the latter weighs observations above $Y_{0}^{P}$ more because of the logarithmic transformation. Finally, the imputation of zero incomes inflates estimates of NL‐IGEs at the low to medium parental income range (the parental income ranges where zero incomes for children are most prevalent).

22 Throughout this section we use a variety of data sources. We discuss these briefly in the main text. Section F in the Online Appendix describes them in detail.

23 See Tables A13 and A14 in the Online Appendix for an overview of the expenditures on education systems in the US, Denmark, and the rest of Scandinavia. They demonstrate the greater generosity of the Danish system measured in a variety of ways. Expenditures on preschools are especially generous. Currie (2001) and Simonsen (2010) give detailed descriptions of expenditures and pricing schemes in early education in the US and Denmark, respectively.

24 The Scandinavian daycare and preschool system is rooted in a social pedagogy tradition as opposed to many other European countries and the US, which focus more on an educational approach (OECD, 2001, 2006). “The English‐speaking world has adopted a ‘readiness for school’ approach, which although defined broadly focuses in practice on cognitive development in the early years, and the acquisition of a range of knowledge, skills, and dispositions. A disadvantage inherent in this approach is the use of programmes and approaches that are poorly suited to the psychology and natural learning strategies of young children. In countries inheriting a social pedagogy tradition (Nordic and Central European countries), the kindergarten years are seen as a broad preparation for life and the foundation stage of lifelong learning.” (OECD, 2006, p. 2). The Nordic approach is summarized as follows. “The core of the curriculum is the dialogue between adult and child and creative activities, discussions and reflections. The curriculum sets goals for early education, but is flexible so that it can be adapted to local and individual needs.” (Taguma *et al*., 2013, Table 2.1). In recent years, however, early childhood care in Denmark has increased its focus on education as well (see Jensen *et al*., 2010, for a discussion).

25 Havnes and Mogstad (2011a,b) show that most of the uptake in publicly provided childcare comes from children who were in informal care arrangements. We return to this point in our conclusion.

26 Currie and Thomas (2000) and Elango *et al*. (2016) are examples.

27 Figures A7 and A8 in the Online Appendix provide similar results for adults using data from the Program for the International Assessment of Adult Competencies (PIAAC) and from the International Adult Literacy Skills Survey (IALS).

28 Roughly 10 percent are born before 1980. Modal years are 1981–1991 where 6.5–8.5 percent of the sample are born each year.

29 Primary school grades are not binding for the child's further educational trajectory for this cohort.

30 See Table A19 in the Online Appendix for sources of loss of sample information.

31 Table A15 in the Online Appendix presents regression coefficients of parental permanent wage income and wealth on children's high school completion and college attendance where we sample cohorts in the Danish register data with the same distribution as observed in the CNLSY data, and where we sample the number of observations in each cohort as observed in the CNLSY data. The results do not differ significantly or qualitatively from our main results, which we present in Table 4.

32 Results are robust to using gross income including Unemployment Insurance benefits (UI) and welfare transfers.

33 These include the value of major owned durables (e.g., housing), as well as debts, but not pension assets.

34 These include valuations of major owned durables.

35 See Browning *et al*. (2013) for a discussion of Danish data.

36 Home equity comprises a larger share of households' net wealth in Denmark than in the US. In 2010, home equity was estimated as approximately 24 percent of households' net wealth in the US (Gottschalck *et al*., 2013). In Denmark in 2014, this was 37.5 percent (Statistics Denmark, 2016). Reported shares for both countries include pension savings in total net wealth.

37 Cameron and Heckman (1993) and Heckman *et al*. (2014) show that these two concepts are not equivalent. However, omitting the GED from the definition of high school completion would likely reduce the similarities of Denmark and the US, as the Danish measure of high school completion also includes a version of the GED (the Højere Forberedelseseksamen (HF) or Higher Preparatory Examination). The HF is a substitute high school degree designed for those who dropped out of high school or earlier educational levels before they completed this. While grade point averages (GPAs) from the HF provide access to further education and university as regular academic high school (Gymnasium) does, HF graduates have lower average levels of completed schooling and lower adult income (likely because HF completion instead of Gymnasium completion, for a given GPA, proxies fewer skills on other dimensions; Heckman and Rubinstein, 2001). Students often take the HF at older ages than normal high school students. Some are high school dropouts while others have not enrolled in high school, but dropped out of education after the compulsory years and have spent 5–10 years out of the educational system.

38 The Danish educational system is rooted in a Northern European tradition and is not directly comparable to the US system, while secondary and tertiary educations in Denmark are highly comparable to those in countries such as Germany and Norway. Our definitions of “high school” and “college” bring the US and Danish system closer, both qualitatively and in population means. However, this simplification of the Danish educational ladder reduces comparability to other Scandinavian schooling systems, unless similar simplifications are made there as well. Figure A52 in the data section of the Online Appendix illustrates how the two schooling outcomes are affected by our definitions and age restrictions in Denmark.

39 Social promotion reduces the academic material needed to pass this level. Social promotion can, in a more complex form, result in reducing the academic levels needed to complete a given education, and thus inflate graduation rates, thereby invalidating cross‐country comparisons of the educational levels in question. The phenomenon exists in both countries, evidenced by the substantial attention it has received in the public debate. For Denmark, see Berligske Tidende (2015), Politiken (2014), and Produktivitetskommissionen (Productivity Commission) (2014) for examples of a discussion from the public debate. For the US, see McCombs *et al*. (2009), President Clinton (1998), and United States Department of Education (1999). However, there are no data available that allow us to test for differences and/or similarities between social promotion in Denmark and the US.

40 In the US, the law dictates that children should attend school until they turn 16–18 (depending upon the state). For most states, this includes the first year of high school. In Denmark, there is no minimum school‐leaving age, but instead a minimum number of years of schooling. Children are not allowed to leave school before they have completed ninth grade. As a consequence, most US children have to begin high school even though they are not forced to complete it. This might induce some to graduate high school who would not have done so in the Danish setting, and thus might increase (perceived) educational mobility in the US.

41 Our measures of non‐cognitive skills in the two countries are clearly not equivalent. The Danish measure of non‐cognitive skills is more related to an orderliness/effort measure while the US measure is related to behavioral problems. Another concern when using grades is that our measures of non‐cognitive skills are more closely related to academic achievement than to socio‐emotional skills. We do not consider this to be an issue in the present case. When we estimate factor loadings and perform variance decompositions from the two factors on outcomes Driving Under the Influence (DUI) and psychiatric admissions, these outcomes are significantly more associated with non‐cognitive (socio‐emotional) skills than cognitive skills. The factor for non‐cognitive skills explains around three to five times as much of the variance in DUI and mental disorders compared to the factor for cognitive skills.

42 Figures A16(a)–(d) in the Online Appendix show the corresponding results for quantiles of parental income and wealth. The overall pattern and conclusions are unchanged, although gradients do change as a result of the distributional differences between income/wealth in Denmark and the US.

43 Figures A17 and A18 in the Online Appendix show rates of high school completion and college attendance by wage income and wage income plus public benefits in levels and ranks, respectively. The figures show that there is little or no difference in the relationship between the two income measures for both levels of schooling in the US and for college attendance in Denmark. For rates of high school completion in Denmark, however, we see that the inclusion of public benefits results in a steeper negative slope at the very lowest levels of incomes. The reason for this anomaly is that a small share of those with close to zero wage earnings and public transfers in Denmark live from capital income or profits from businesses instead. The educational outcomes of this group are very different from those experienced by children from the remaining low‐income families.

44 Table A16 in the Online Appendix shows the corresponding regression coefficients for parental income and wealth ranks for children's high school completion and college attendance controlling for child skills at age 15, family background, and school quality.

45 Family background variables: child gender (0/1), immigrant/minority (0/1), urban region (0/1), siblings, mother's age at birth, and mother's years of schooling. School characteristics for US include: grade for how teachers care about students, grade for whether school is considered safe, a dummy for whether a child feels peer pressure to work hard, a dummy for whether a child feels peer pressure to skip school, a dummy for whether a child has received sex education in school, and a dummy for whether a child attends private school. School characteristics for Denmark include: for each school, the means of previous cohort's mother's age at birth, mother's high school completion, and mother's college attendance, high school completion, and college attendance.

46 Parental background variables are: child gender (0/1), immigrant/minority (0/1), urban region (0/1), number of siblings, mother's age at birth, and mother's years of schooling.

47 See OECD (2014) for an overview of public and private resources devoted to primary, secondary, and tertiary education, and Table A13 in the Online Appendix.

48 As data sources from the two countries differ (Denmark has register data and the US has survey data), we proxy school characteristics by aspects that are not directly comparable. For the US, we use parents' ratings of their child's school; for Denmark, we use average characteristics of earlier cohorts in a given school. The results are used to illustrate that substantial sorting takes place in both Denmark and the US and that this sorting coincides with parental resources. Hence, once we also condition on our school characteristics measures, the income and wealth gradients in children's schooling are reduced further. Importantly, the results are not causal, nor do they identify the impact of school quality on later education.

49 Figure A21 in the Online Appendix shows the equivalent results using wage earnings and not gross income including transfers. Figures A22 and A23 show non‐linear estimates of children's education (high school completion, college graduation, master degree, highest grade completed) on parents' average highest grade completed. The figures show non‐linearities across different levels of parental education and differences between the various measures of children's education. However, the figures show no patterns that favor educational mobility in Denmark over that in the US.

50 See the summary of the literature in Heckman and Mosso (2014) and Lochner and Monge‐Naranjo (2016).

51 Cameron and Taber (2004), Carneiro and Heckman (2002), and Keane and Wolpin (2001) find little evidence of this relationship as opposed to Bailey and Dynarski (2011), Belley and Lochner (2007), and Lochner and Monge‐Naranjo (2012), who report stronger evidence of credit constraints to, for example, college enrollment.

52 This is consistent with the evidence reported in Cameron and Heckman (2001).

53 This finding mirrors the analysis of Heckman (2011) in showing the importance of cognitive and non‐cognitive skills in explaining minority–majority differences in college attainment in the US.

54 In this analysis we use the data that were introduced in the *Data* subsection of Section I..

55 Eligibility for unemployment insurance benefits is based on previous employment and membership of an unemployment insurance fund (insurance is tax‐deductable and benefits are subsidized by the public sector). The figure reports the maximum level available. Below this level, unemployment insurance benefits replace wage earnings by a rate of 90 percent. Social assistance is means‐tested (on household level) such that income earned is deducted 1:1 from benefits. Levels differ by whether recipients have children or not.

56 See https://www.retsinformation.dk/Forms/R0710.aspx?id=53834, https://www.retsinformation.dk/Forms/R0710.aspx?id=53848, and https://www.retsinformation.dk/Forms/R0710.aspx?id=53886 (accessed April 1, 2016) for the specific legislation in question.

57 Jonassen (2013) studies behavioral responses around age 25. He finds that take‐up of social assistance increases substantially once benefits increase to full level at age 25. Furthermore, he finds no evidence of substitution between social assistance and public education grant at age 25. This contrast to our results suggests that the link between social assistance and education is mainly present at lower‐tier educations (i.e., for low‐skilled individuals), which would have been completed at age 25 in any case.

58 Heckman and Mosso (2014) present evidence supporting disequalization.

59 Cunha and Heckman (2007), Carneiro *et al*. (2013), and Heckman and Mosso (2014) discuss static and dynamic complementarity. Static complementarity is described by the “Matthew effect”: *to those who have, more is given*. Dynamic complementarity is the effect of investments at one age on building complementarity at later ages.

60 Danish schools receive higher rates per student for special needs education, but not based on overall resource level of catchment area. See the Public School Law (https://www.retsinformation.dk/Forms/r0710.aspx?id=163970).

61 A vast literature has described the significant impacts of school and peer quality on educational outcomes and income. See Sacerdote (2011) for examples.

62 See Black and Machin (2011) for a review of this literature. Tiebout (1956) and Black (1999) show that housing prices are affected by school quality. More related to the Danish case, Machin and Salvanes (2016) provide recent evidence on this issue from Norway.

63 In a similar vein, in our analysis of income mobility, we have chosen an age range to measure income that should proxy lifetime income closely. Yet we still only provide a snapshot of income mobility under the assumption of homogeneous time preferences. Educational attainment and time preference correlate (Oreopoulos and Salvanes, 2011). As a consequence, intergenerational income mobility estimates reported here and in the remaining literature might be biased if income at older ages should receive less weight than income at early ages for individuals without college degrees, and vice versa. One possible next step would be to create a generalized intergenerational income elasticity, which evaluates the entire stream of lifetime earnings for parents and children, allowing for differential time preferences. Even better would be to form the value functions of lifetime earnings (e.g., Hai and Heckman, 2016).

64 See the discussion in Lochner and Monge‐Naranjo (2016).

65 See Section D of the Online Appendix for a brief outline.

66 See Section E of the Online Appendix for a brief introduction and examples.

## Appendix 1

### 1.1

**Table A1:** Summary from the previous literature of IGE estimates and income definitions for Denmark and the US

Child's income	Parental income	Income definition	IGE estimate
**Munk *et al*. (** 2016 **), for Denmark, from Register data, Statistics Denmark**			
Log(Son's earnings), measured in 2004–2008, age 7–14 in 1980	Log(Father's earnings), measured in 1980–1984	Income definition not detailed in paper	0.171 (0.002)
Log(Son's income), measured in 2004–2008, age 7–14 in 1980	Log(Father's income), measured in 1980–1984	Earnings, capital income, income transfers	0.241 (0.002)
**Chetty *et al*. (** 2014 **), for US, from Federal income tax records**			
Log(Child's family income), measured in 2011–2012, cohorts born in 1980–1982	Log(Parental family income), when children were 10 and 16	Total pre‐tax income at the household level from all sources, excluding nontaxable cash benefits	Sons, 0.349 (0.001) Daughters, 0.342 (0.001) Imputing 0 income with $1: Sons, 0.697 (0.001) Daughters, 0.540 (0.001)
**Blanden *et al*. (** 2014 **), for US, from PSID**			
Log(Son's earnings), measured at ages 30 and 34	Log(Parental family income), measured when children were 10 and 16	Child's earnings; parental income from all sources	0.385 (0.046)
**Hussain *et al*. (** 2009 **), for Denmark, from Register data, Statistics Denmark**			
Log(Son's earnings), measured in 2002, at ages 30–40	Log(Father's earnings), measured in 1984–1988, at ages 30–66	Wage rate multiplied by hours of work plus sickness pay and unemployment insurance benefits	0.136 (0.004); when using “measures as in other studies” (see text): 0.05–0.11
**Bratsberg *et al*. (** 2007 **), for Denmark, from Register data, Statistics Denmark**			
Log(Son's Earnings), measured in 1998 and 2000, cohort 1958	Log(Father's earnings), measured in 1980–1981	Total earnings from all employers	0.121 (0.008)
Log(Son's Earnings), measured in 1998 and 2000, cohort 1958	Log(Father's earnings), measured in 1980–1981	Sum of mother's and father's earnings	0.151 (0.009)
**Bratsberg *et al*. (** 2007 **), for US, from NLSY**			
Log(Son's Earnings), measured in 1995 and 2001, cohort 1957–1964	Log(Family income), measured in 1978–1979	Family income from all sources	0.542 (0.008)
**Jäntti *et al*. (** 2006 **), for Denmark, from Register data, Statistics Denmark**			
Log(Child's earnings), measured in 1998 and 2002, cohorts 1958–1960	Log(Father's earnings), measured in 1980	Wages, salaries, and self‐employment income	Sons, 0.071 [0.064,0.079] Daughters, 0.034 [0.027,0.041]
**Jäntti *et al*. (** 2006 **), for US, from NLSY**			
Log(Child's earnings), measured in 1995 and 2001, cohorts 1957–1964	Log(Father's earnings), measured in 1978	Family income from all sources	Sons, 0.517 [0.444,0.590] Daughters, 0.283 [0.181,0.385]
**Mazumder (** 2005 **), for US, from SIPP matched to SSA**			
Log(Child's earnings), measured in 1995–1998, for sons born in 1963–1968	Log(Father's earnings), measured in 1970–1985	Individual's annual taxable earnings from Social Security Administration's records	Sons, Tobit 0.61 (0.096) Daughters, Tobit: 0.570 (0.159)
**Bonke *et al*. (** 2005 **), for Denmark, from Register data, Statistics Denmark**			
Log(Child's earnings), measured in 2002, ages 30–40	Log(Parental earnings), measured in 1980–1984	Earnings defined as hourly wage multiplied by annual hours worked	0.240 (0.096) controlling for father's age
**Eriksson *et al*. (** 2005 **), for Denmark, from Register data, Statistics Denmark**			
Log(Child's earnings), measured in 2001, cohort of 1954	Log(Father's earnings), measured in 1968 or 1969	Earnings defined as wage income rounded to seven intervals for fathers and 11 intervals for children	Sons, 0.292 (0.077) Daughters, 0.210 (0.065) controlling for father's age
**Chadwick and Solon (** 2002 **), for US, from PSID**			
Log(Child's family income), measured in 1991, born in 1951–1966	Log(Family income) measured in 1967–1971	Total taxable income from all sources for all family members	Sons, 0.535 (0.059) Daughters, 0.429 (0.063)
**Björklund and Jäntti (** 1997 **), for US, from PSID**			
Log(Son's earnings), measured in 1987, cohorts 1951–1959	Log(Father's earnings) measured in 1967–1971	Total annual earnings from wages and salaries	0.392 (0.082)
Log(Son's earnings), measured in 1987, cohorts 1951–1959	Log(Father's predicted earnings) measured in 1967–1971	Predicted from education and occupation	IV, 0.516 (0.138) TSIV, 0.52 (0.14)
**Solon (** 1992 **), for US, from PSID**			
Log(Son's earnings), measured in 1984, ages 25–33	Log(Father's earnings), measured in 1967–1972	Total annual earnings from wages and salaries	0.413 (0.09)
Log(Son's hourly wage), measured in 1984, ages 25–33	Log(Father's hourly wage) measured in 1967	Hourly wage: ratio of annual earnings to annual hours of work	0.294 (0.052) IV, 0.449 (0.095)
Log(Son's family income), measured in 1984, ages 25–33	Log(Family income) measured in 1967	Total taxable income from all sources for all family members	0.483 (0.069) IV, 0.530 (0.123)
**Zimmerman (** 1992 **), for US, from NLS**			
Log(Son's earnings), measured in 1981, ages 29–39	Log(Father's earnings), measured in 1965–1970	Total annual earnings from wages and salaries	0.54 (0.08)

*Notes*: This table summarizes the results from the previous literature of estimates of the intergenerational income elasticity for Denmark and the US (selected studies only for the US). The table summarizes the data sources used, income types compared, cohorts and ages at which income is measured, the income definitions used, and each paper's main estimates (OLS log–log, unless otherwise noted).

## References

[sjoe12219-bib-0001] Aaberge, R. , Wennemo, T. , Björklund, A. , Jäntti, M. , Pedersen, P. J. , and Smith, N. (2000), Unemployment Shocks and Income Distribution: How did the Nordic Countries Fare During Their Crises?, The Scandinavian Journal of Economics 102, 77–99.

[sjoe12219-bib-0002] Aaberge, R. , Björklund, A. , Jäntti, M. , Palme, M. , Pedersen, P. J. , Smith, N. , and Wennemo, T. (2002), Income Inequality and Income Mobility in the Scandinavian Countries Compared to the United States, Review of Income and Wealth 48, 443–469.

[sjoe12219-bib-0004] Baily, M. N. (2016), What Sanders Gets Right and Wrong About Denmark, Online article, Inside Sources, http://www.insidesources.com/what‐sanders‐gets‐right‐and‐wrong‐about‐denmark/.

[sjoe12219-bib-0003] Bailey, M. J. and Dynarski, S. M. (2011), Gains and Gaps: Changing Inequality and U.S. College Entry and Completion, NBER Working Paper 17633.

[sjoe12219-bib-0005] Belley, P. and Lochner, L. (2007), The Changing Role of Family Income and Ability in Determining Educational Achievement, Journal of Human Capital 1, 37–89.

[sjoe12219-bib-0006] Berligske Tidende (2015), Studerende består, selvom de burde dumpe, http://www.b.dk/nationalt/studerende‐bestaar‐selvom‐de‐burde‐dumpe.

[sjoe12219-bib-0007] Björklund, A. and Jäntti, M. (1997), Intergenerational Income Mobility in Sweden Compared to the United States, American Economic Review 87(5), 1009–1018.

[sjoe12219-bib-0008] Björklund, A. and Jäntti, M. (2011), Intergenerational Income Mobility and the Role of Family Background, in Salverda W., Nolan B., and Smeeding T. M. (eds), Oxford Handbook of Economic Inequality, Chapter 20, Oxford University Press, Oxford, 491–521.

[sjoe12219-bib-0009] Björklund, A. , Roine, J. , and Waldenström, D. (2012), Intergenerational Top Income Mobility in Sweden: Capitalist Dynasties in the Land of Equal Opportunity?, Journal of Public Economics 96, 474–484.

[sjoe12219-bib-0010] Black, S. E. (1999), Do Better Schools Matter? Parental Valuation of Elementary Education, Quarterly Journal of Economics 114, 577–599.

[sjoe12219-bib-0011] Black, S. E. and Devereux, P. J. (2011), Recent Developments in Intergenerational Mobility, in Ashenfelter O. C. and Card D. (eds), Handbook of Labor Economics, Volume 4, Part B, Chapter 16, Elsevier, Amsterdam, 1487–1541.

[sjoe12219-bib-0012] Black, S. E. and Machin, S. (2011), Housing Valuations of School Performance, in Hanushek E. A., Machin S., and Woessmann L. (eds.), Handbooks of the Economics of Education, Volume 3, Chapter 10, Elsevier, Amsterdam, 485–519.

[sjoe12219-bib-0013] Blanden, J. (2013), Cross‐Country Rankings in Intergenerational Mobility: A Comparison of Approaches from Economics and Sociology, Journal of Economic Surveys 27, 38–73.

[sjoe12219-bib-0014] Blanden, J. , Haveman, R. , Smeeding, T. , and Wilson, K. (2014), Intergenerational Mobility in the United States and Great Britain: A Comparative Study of Parent–Child Pathways, Review of Income and Wealth 60, 425–449. 30111896 10.1111/roiw.12032 PMC6089545

[sjoe12219-bib-0015] Bonke, J. , Hussain, M. A. , and Munk, M. D. (2005), A Comparison of Danish and International Findings on Intergenerational Earnings Mobility, Working Paper, Social Forsknings Instituttet.

[sjoe12219-bib-0016] Borghans, L. , Golsteyn, B. H. H. , Heckman, J. J. , and Humphries, J. E. (2011a), Identification Problems in Personality Psychology, Personality and Individual Differences 51(3), 315–320. 21731170 10.1016/j.paid.2011.03.029 PMC3126096

[sjoe12219-bib-0017] Borghans, L. , Golsteyn, B. H. H. , Heckman, J. J. , and Humphries, J. E. (2011b), IQ, Achievement, and Personality, Unpublished manuscript, University of Maastricht and University of Chicago (revised from the 2009 version).

[sjoe12219-bib-0094] Borghans, L. , Golsteyn, B. H. H. , Heckman, J. J. , and Humphries, J. E. (2016), What Do Grades and Achievement Tests Measure?, Proceedings of the National Academy of Sciences, submitted. 10.1073/pnas.1601135113 PMC5127298 27830648

[sjoe12219-bib-0018] Boserup, S. , Kopczuk, W. , and Kreiner, C. T. (2013), Intergenerational Wealth Mobility: Evidence from Danish Wealth Records of Three Generations, Dissertation chapter, University of Copenhagen.

[sjoe12219-bib-0019] Bratsberg, B. , Røed, K. , Raaum, O. , Naylor, R. , Jäntti, M. , Eriksson, T. , and Österbacka, E. (2007), Nonlinearities in Intergenerational Earnings Mobility: Consequences for Cross‐Country Comparisons, Economic Journal 117(519), C72–C92.

[sjoe12219-bib-0020] Browning, M. , Gørtz, M. , and Leth‐Petersen, S. (2013), Housing Wealth and Consumption: A Micro Panel Study, Economic Journal 123(568), 401–428.

[sjoe12219-bib-0021] Cameron, S. V. and Heckman, J. J. (1993), The Nonequivalence of High School Equivalents, Journal of Labor Economics 11, 1–47.

[sjoe12219-bib-0022] Cameron, S. V. and Heckman, J. J. (2001), The Dynamics of Educational Attainment for Black, Hispanic, and White Males, Journal of Political Economy 109, 455–499.

[sjoe12219-bib-0023] Cameron, S. V. and Taber, C. (2004), Estimation of Educational Borrowing Constraints Using Returns to Schooling, Journal of Political Economy 112, 132–182.

[sjoe12219-bib-0024] Carneiro, P. and Heckman, J. J. (2002), The Evidence on Credit Constraints in Post‐Secondary Schooling, Economic Journal 112(482), 705–734.

[sjoe12219-bib-0025] Carneiro, P. , Meghir, C. , and Parey, M. (2013), Maternal Education, Home Environments, and the Development of Children and Adolescents, Journal of the European Economic Association 11, 123–160.

[sjoe12219-bib-0026] Cascio, E. U. (2009), Do Investments in Universal Early Education Pay Off? Long‐Term Effects of Introducing Kindergartens into Public Schools, NBER Working Paper 14951.

[sjoe12219-bib-0027] Chadwick, L. and Solon, G. (2002), Intergenerational Income Mobility among Daughters, American Economic Review 92(1), 335–344.

[sjoe12219-bib-0028] Chetty, R. , Hendren, N. , Kline, P. , and Saez, E. (2014), Where is the Land of Opportunity? The Geography of Intergenerational Mobility in the United States, Quarterly Journal of Economics 129, 1553–1623.

[sjoe12219-bib-0029] Chevalier, A. , Harmon, C. , O'Sullivan, V. , and Walker, I. (2013), The Impact of Parental Income and Education on the Schooling of their Children, IZA Journal of Labor Economics 2, 1–22.

[sjoe12219-bib-0030] Corak, M. (2006), Do Poor Children Become Poor Adults? Lessons from a Cross Country Comparison of Generational Earnings Mobility, Institute for the Study of Labor (IZA) Discussion Paper 1993.

[sjoe12219-bib-0031] Corak, M. (2013), Income Inequality, Equality of Opportunity, and Intergenerational Mobility, Journal of Economic Perspectives 27, 79–102.

[sjoe12219-bib-0032] Cunha, F. and Heckman, J. J. (2007), The Technology of Skill Formation, American Economic Review 97(2), 31–47.

[sjoe12219-bib-0033] Cunha, F. and Heckman, J. J. (2008), Formulating, Identifying and Estimating the Technology of Cognitive and Noncognitive Skill Formation, Journal of Human Resources 43, 738–782. 10.3982/ECTA6551 PMC2885826 20563300

[sjoe12219-bib-0034] Currie, J. (2001), Early Childhood Education Programs, Journal of Economic Perspectives 15, 213–238.

[sjoe12219-bib-0035] Currie, J. and Thomas, D. (2000), School Quality and the Longer‐Term Effects of Head Start, Journal of Human Resources 35, 755–774.

[sjoe12219-bib-0036] Dahl, M. and DeLeire, T. (2008), The Association between Children's Earnings and Father's Lifetime Earnings: Estimates using Administrative Data, Institute for Research on Poverty, University of Wisconsin‐Madison, Discussion Paper 1342‐08.

[sjoe12219-bib-0037] Datta Gupta, N. and Simonsen, M. (2010), Non‐Cognitive Child Outcomes and Universal High Quality Child Care, Journal of Public Economics 94, 30–43.

[sjoe12219-bib-0038] Datta Gupta, N. and Simonsen, M. (2012), The Effects of Type of Non‐Parental Child Care on Pre‐Teen Skills and Risky Behavior, Economics Letters 116, 622–625.

[sjoe12219-bib-0039] Davies, J. B. , Zhang, J. , and Zeng, J. (2005), Intergenerational Mobility Under Private vs. Public Education, The Scandinavian Journal of Economics 107, 399–417.

[sjoe12219-bib-0040] Edin, P.‐A. and Topel, R. (1997), Wage Policy and Restructuring: The Swedish Labor Market Since 1960, in Freeman R. B., Topel R., and Swedenborg B. (eds.), The Welfare State in Transition: Reforming the Swedish Model, Chapter 4, University of Chicago Press, Chicago, 155–202.

[sjoe12219-bib-0041] Elango, S. , Hojman, A. , García, J. L. , and Heckman, J. J. (2016), Early Childhood Education, in Moffitt R. (ed.), Economics of Means‐Tested Transfer Programs in the United States, Volume II, Chapter 4, University of Chicago Press, Chicago.

[sjoe12219-bib-0042] Eriksson, T. , Bratsberg, B. , and Raaum, O. (2005), Earnings Persistence Across Generations: Transmission Through Health?, Memoradum No. 35, Department of Economics, University of Oslo.

[sjoe12219-bib-0043] Forslund, A. and Krueger, A. B. (1997), An Evaluation of the Swedish Active Labor Market Policy: New and Received Wisdom, in Freeman R. B., Topel R., and Swedenborg B. (eds.), The Welfare State in Transition: Reforming the Swedish Model, Chapter 6, University of Chicago Press, Chicago, 267–298.

[sjoe12219-bib-0044] Fredriksson, P. and Topel, R. (2010), Wage Determination and Employment in Sweden Since the Early 1990s: Wage Formation in a New Setting, in Freeman R. B., Swedenborg B., and Topel R. H. (eds.), Reforming the Welfare State: Recovery and Beyond in Sweden, Chapter 3, University of Chicago Press, Chicago, 83–126.

[sjoe12219-bib-0045] Freeman, R. B. , Swedenborg, B. , and Topel, R. (2010), Introduction, in Freeman R. B., Swedenborg B., and Topel R. H. (eds.), Reforming the Welfare State: Recovery and Beyond in Sweden, University of Chicago Press, Chicago, 1–23.

[sjoe12219-bib-0046] Gottschalck, A. , Vornovytskyy, M. , and Smith, A. (2013), Household Wealth and Debt in the U.S.: 2000 to 2011, Press release, US Census Bureau, Washington, DC.

[sjoe12219-bib-0047] Hai, R. and Heckman, J. J. (2016), Inequality in Human Capital and Endogenous Credit Constraints, Review of Economic Dynamics, under revision. 10.1016/j.red.2017.01.001 PMC5476319 28642641

[sjoe12219-bib-0048] Harmon, C. , Oosterbeek, H. , and Walker, I. (2003), The Returns to Education: Microeconomics, Journal of Economic Surveys 17, 115–155.

[sjoe12219-bib-0049] Havnes, T. and Mogstad, M. (2011a), Money for Nothing? Universal Child Care and Maternal Employment, Journal of Public Economics 95, 1455–1465.

[sjoe12219-bib-0050] Havnes, T. and Mogstad, M. (2011b), No Child Left Behind: Subsidized Child Care and Children's Long‐Run Outcomes, American Economic Journal: Economic Policy 3(2), 97–129.

[sjoe12219-bib-0051] Heckman, J. J. (2011), The American Family in Black and White: A Post‐Racial Strategy for Improving Skills to Promote Equality, Daedalus 140, 70–89. 22605880 10.1162/DAED_a_00078 PMC3351134

[sjoe12219-bib-0053] Heckman, J. J. and Mosso, S. (2014), The Economics of Human Development and Social Mobility, Annual Review of Economics 6, 689–733. 10.1146/annurev-economics-080213-040753 PMC4204337 25346785

[sjoe12219-bib-0054] Heckman, J. J. and Rubinstein, Y. (2001), The Importance of Noncognitive Skills: Lessons from the GED Testing Program, American Economic Review 91(2), 145–149.

[sjoe12219-bib-0055] Heckman, J. J. , Stixrud, J. , and Urzúa, S. (2006), The Effects of Cognitive and Noncognitive Abilities on Labor Market Outcomes and Social Behavior, Journal of Labor Economics 24, 411–482.

[sjoe12219-bib-0052] Heckman, J. J. , Humphries, J. E. , and Kautz, T. (eds.) (2014), The Myth of Achievement Tests: The GED and the Role of Character in American Life, University of Chicago Press, Chicago.

[sjoe12219-bib-0056] Hertz, T. , Jayasundera, T. , Piraino, P. , Selcuk, S. , Smith, N. , and Verashchagina, A. (2008), The Inheritance of Educational Inequality: International Comparisons and Fifty‐Year Trends, B.E. Journal of Economic Analysis and Policy 7, 1–46.

[sjoe12219-bib-0057] Hussain, M. , Munk, M. D. , and Bonke, J. (2009), Intergenerational Earnings Mobilities – How Sensitive Are They to Income Measures?, Journal of Income Distribution 18, 79–92.

[sjoe12219-bib-0058] Jäntti, M. , Røed, K. , Naylor, R. , Björklund, A. , Bratsberg, B. , Raaum, O. , Österbacka, E. , and Eriksson, T. (2006), American Exceptionalism in a New Light: A Comparison of Intergenerational Earnings Mobility in the Nordic Countries, the United Kingdom and the United States, Institute for the Study of Labor (IZA) Discussion Paper 1938.

[sjoe12219-bib-0059] Jensen, A. S. , Broström, S. , and Hansen, O. H. (2010), Critical Perspectives on Danish Early Childhood Education and Care: Between the Technical and the Political, Early Years: An International Research Journal 30, 243–254.

[sjoe12219-bib-0060] Jonassen, A. B. (2013), Disincentive Effects of a Generous Social Assistance Scheme, SFI Working Paper WP 01:2013, Danish National Centre for Social Research, Copenhagen.

[sjoe12219-bib-0061] Keane, M. P. and Wolpin, K. I. (2001), The Effect of Parental Transfers and Borrowing Constraints on Educational Attainment, International Economic Review 42, 1051–1103.

[sjoe12219-bib-0062] Krueger, A. B. (2012), The Rise and Consequences of Inequality in the United States, Speech given at the Center for American Progress, Washington DC.

[sjoe12219-bib-0063] Lochner, L. J. and Monge‐Naranjo, A. (2012), Credit Constraints in Education, Annual Review of Economics 4, 225–256.

[sjoe12219-bib-0064] Lochner, L. J. and Monge‐Naranjo, A. (2016), Student Loans and Repayment: Theory, Evidence and Policy, NBER Working Paper 20849.

[sjoe12219-bib-0065] McCombs, J. S. , Kirby, S. N. , and Mariano, L. T. (eds.) (2009), Ending Social Promotion Without Leaving Children Behind: The Case of New York City, RAND Corporation, Santa Monica, CA.

[sjoe12219-bib-0066] Machin, S. and Salvanes, K. G. (2016), Valuing School Quality via a School Choice Reform, The Scandinavian Journal of Economics 118, 3–24.

[sjoe12219-bib-0067] Mazumder, B. (2005), Fortunate Sons: New Estimates of Intergenerational Mobility in the United States Using Social Security Earnings Data, Review of Economics and Statistics 87, 235–255.

[sjoe12219-bib-0068] Munk, M. D. , Bonke, J. , and Hussain, M. A. (2016), Intergenerational Top Income Persistence: Denmark Half the Size of Sweden, Economics Letters 140, 31–33.

[sjoe12219-bib-0069] Nybom, M. and Stuhler, J. (2015), Biases in Standard Measures of Intergenerational Income Dependence, Institute for Evaluation of Labour Market and Education Policy, Working Paper 2015:13, Uppsala, Sweden.

[sjoe12219-bib-0070] Oreopoulos, P. and Salvanes, K. G. (2011), Priceless: The Nonpecuniary Benefits of Schooling, Journal of Economic Perspectives 25, 159–184.

[sjoe12219-bib-0071] OECD (2001), Executive Summary: Starting Strong – Early Education, Technical Report, OECD, Paris.

[sjoe12219-bib-0072] OECD (2004), Learning for Tomorrow's World: First Results from PISA 2003, Technical Report, OECD, Paris.

[sjoe12219-bib-0073] OECD (2006), Executive Summary: Starting Strong II – Early Childhood Education and Care, Technical Report, OECD, Paris.

[sjoe12219-bib-0074] OECD (2014), Education at a Glance 2014: OECD Indicators, Technical Report, OECD, Paris.

[sjoe12219-bib-0075] Pedersen, P. J. and Smith, N. (2000), Trends in Danish Income Distribution, LABOUR 14, 523–546.

[sjoe12219-bib-0076] Politiken (2014), Rektor: Taxameter sænker barren til eksamen, http://politiken.dk/indland/uddannelse/ECE2465532/rektor‐taxameter‐saenker‐barren‐til‐eksamen/.

[sjoe12219-bib-0077] President Clinton (1998), State of the Union Address (available at http://www.washingtonpost.com/wp‐srv/politics/special/states/docs/sou98.htm).

[sjoe12219-bib-0078] Produktivitetskommissionen (Productivity Commission) (2014), Det handler om velstand og velfærd, Slutrapport (final report), Produktivitetskommissionen, Copenhagen.

[sjoe12219-bib-0079] Rosen, S. (1997), Public Employment, Taxes, and the Welfare State in Sweden, in Freeman R. B., Topel R., and Swedenborg B. (eds.), The Welfare State in Transition: Reforming the Swedish Model, Chapter 2, University of Chicago Press, Chicago, 79–108.

[sjoe12219-bib-0080] Sacerdote, B. (2011), Nature and Nurture Effects on Children's Outcomes: What Have we Learned from Studies of Twins and Adoptees?, in Benhabib J., Bisin A., and Jackson M. O. (eds.), Handbook of Social Economics, Volume 1A, Chapter 1, Elsevier, Amsterdam, 1–30.

[sjoe12219-bib-0081] Sanders, B. (2013), What Can We Learn from Denmark?, Blog post, Huffington Post (http://www.huffingtonpost.com/rep‐bernie‐sanders/what‐can‐we‐learn‐from‐de_b_3339736.html).

[sjoe12219-bib-0082] Simonsen, M. (2010), Price of High‐Quality Daycare and Female Employment, The Scandinavian Journal of Economics 112, 570–594.

[sjoe12219-bib-0083] Solon, G. (1992), Intergenerational Income Mobility in the United States, American Economic Review 82(3), 393–408.

[sjoe12219-bib-0084] Solon, G. (2002), Cross‐Country Differences in Intergenerational Earnings Mobility, Journal of Economic Perspectives 16, 59–66.

[sjoe12219-bib-0085] Solon, G. (2004). A Model of Intergenerational Mobility Variation over Time and Place, in Corak M. (ed.), Generational Income Mobility in North America and Europe, Chapter 2, Cambridge University Press, Cambridge, 38–47.

[sjoe12219-bib-0086] Statistics Denmark (2016), Net Wealth by Component and Family Type, http://statistikbanken.dk/statbank5a/default.asp?w=375 (accessed May 6, 2016).

[sjoe12219-bib-0087] Taguma, M. , Litjens, I. , and Makowiecki, K. (2013), Quality Matters in Early Childhood Education and Care: Sweden, Technical Report, OECD, Paris.

[sjoe12219-bib-0088] Tiebout, C. M. (1956), A Pure Theory of Local Expenditures, Journal of Political Economy 64, 416–424.

[sjoe12219-bib-0089] Tranæs, T. (2006), Velfærd og arbejde, in Tranæs T. (ed.), Skat, Arbejde og Lighed, Chapter 1, Gyldendal, Copenhagen, 13–32.

[sjoe12219-bib-0090] Trivedi, P. K. and Zimmer, D. M. (2007), Copula Modeling: An Introduction for Practitioners, Foundations and Trends in Econometrics 1, 1–111.

[sjoe12219-bib-0091] United States Department of Education (1999), Taking Responsibility for Ending Social Promotion: A Guide for Educators and State and Local Leaders, Technical Report, United States Department of Education, Washington, DC.

[sjoe12219-bib-0092] Washington's Blog (2014), The American dream has moved to Scandinavia, http://www.washingtonsblog.com/2014/11/american‐dream‐moved‐scandinavia.html.

[sjoe12219-bib-0093] Zimmerman, D. J. (1992), Regression Toward Mediocrity in Economic Stature, American Economic Review 82(3), 409–429.

